# Geometric stability of topological lattice phases

**DOI:** 10.1038/ncomms9629

**Published:** 2015-11-04

**Authors:** T. S. Jackson, Gunnar Möller, Rahul Roy

**Affiliations:** 1 Department of Physics and Astronomy, University of California at Los Angeles, 475 Portola Plaza, Los Angeles, California 90095, USA; 2 TCM Group, Cavendish Laboratory, J.J. Thomson Avenue, Cambridge CB3 0HE, UK

## Abstract

The fractional quantum Hall (FQH) effect illustrates the range of novel phenomena which can arise in a topologically ordered state in the presence of strong interactions. The possibility of realizing FQH-like phases in models with strong lattice effects has attracted intense interest as a more experimentally accessible venue for FQH phenomena which calls for more theoretical attention. Here we investigate the physical relevance of previously derived geometric conditions which quantify deviations from the Landau level physics of the FQHE. We conduct extensive numerical many-body simulations on several lattice models, obtaining new theoretical results in the process, and find remarkable correlation between these conditions and the many-body gap. These results indicate which physical factors are most relevant for the stability of FQH-like phases, a paradigm we refer to as the geometric stability hypothesis, and provide easily implementable guidelines for obtaining robust FQH-like phases in numerical or real-world experiments.

## Abstract (web summary)

Fractional Chern insulators, topological insulators with partially filled bands, do not require large magnetic fields to form fractional quantum Hall states. Here, the authors investigate the correlation between the stability of such states and the bandgap to their creation on a variety of lattices.

## Graphical abstract

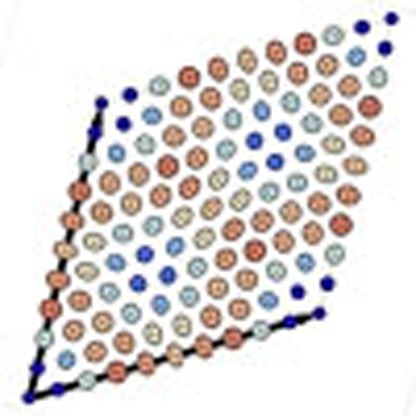

The fractional quantum Hall effect (FQHE) provides a spectacular manifestation of the breakdown of the spin-statistics relation in two dimensions: one obtains quantum number fractionalization^1^,^2^,^3^,^4^,^5^ and, potentially, non-Abelian statistics^6^ which can form the substrate for topologically robust quantum computing^7^. Progress has been hampered by the considerable experimental difficulties involved in realizing the FQHE in the usual setting of a semiconductor heterostructure, but a flurry of interest in the field was set off by the recent insight^8^,^9^,^10^ that these exotic phases of matter may also arise in topologically non-trivial insulators with partially filled flat bands, or fractional Chern insulators^11^ (FCIs). The attractiveness of FCIs stems from the fact that the bandgap *Δ* may be set without the use of a large external magnetic field, the strength of which is one of the limiting factors in the semiconductor FQHE. There are currently a range of experimental proposals for realizing FCIs in cold atom systems^12^,^13^,^14^,^15^,^16^, transition metal oxides^17^,^18^,^19^ and elsewhere; a successful experimental implementation in ultracold fermions was recently announced in ref. 20.

Moreover, FCIs raise theoretical questions independent of their experimental interest. The majority of theoretical work on the FQHE over its 30-year history has focused on the influence of interactions on the Landau level Hamiltonian, which occupies a unique, highly symmetric point in the space of single-particle Hamiltonians. FCI phenomena constitute a non-trivial and poorly understood generalization of the FQHE in which lattice effects are non-negligible; a generic FCI does not have the FQHE as a continuum limit, and examples of lattice effects without any continuum analogue have already been noted^21^,^22^. A generalization of our theoretical understanding of the FQHE to cover the case of FCIs is hence both non-trivial and experimentally relevant.

One possible approach to the stability of FCIs is via the single-mode approximation used by Girvin *et al.*^23^,^24^ (GMP), who made the ansatz that the most relevant excitations which destabilize an FQH ground state are neutral magnetoroton modes generated by the action of electron density operators projected to the lowest Landau level. These operators do not commute with each other, due to the projection, but GMP found that the set of operators remains a closed algebra under commutation. Intuitively, one expects that the form of this algebra plays a crucial role in the stability of the FQHE phases by limiting the set of possible destabilizing interactions. In a generic FCI, however, the analogous set of band-projected density operators is not a closed algebra, nor do the projected densities span the space of single-particle operators^25^: there is no canonical mapping between a general lattice FCI and the continuum FQHE.

In previous work, one of us^26^ derived sufficient conditions for the band-projected density operators in an FCI to satisfy a closed algebra isomorphic to that present in the FQHE, which justified and elaborated upon a heuristic criterion used in previous FCI literature. Quantities describing the geometry of the Chern band (its embedding in Hilbert space) enter this analysis in a natural way as coefficients of terms which must necessarily vanish to obtain a closed algebra; remarkably, only three conditions need to be placed on the band's geometry for the isomorphism to be present to all orders in a long-wavelength expansion. Heuristically, one might expect that reproducing the density operator algebra would then suffice to reproduce the full physics of the FQHE, but this argument has not been fully tested in the literature.

In the present work, we report the results of extensive numerical simulations which demonstrate that quantitative measures based on the band-geometric conditions of ref. 26 are robustly correlated with the many-body gap in realizations of FQH-type phases in different FCI lattice models. In addition to numerical data, we obtain several theoretical results, such as a scaling relation between the gap of an FQH-like state and the number of bands in an FCI model, which is essential for comparing different models. We find that the Berry curvature was computed incorrectly in a number of prior references; in Supplementary Note 1, we discuss why this quantity is defined unambiguously. The remarkably high degree of correlation we find between band geometry and the many-body gap leads us to propose a geometric stability hypothesis: that the algebra of band-projected density operators governs FQH-type phenomena in FCIs, even when the isomorphism does not hold exactly, and that the single-particle conditions investigated here are accurate qualitative estimators of the stability of an FQH-like state. This frames the theoretical problem of generalizing results on the FQHE to cover FCI physics by distilling the effects of the lattice into a small number of quantities measuring the relevant deviations of an FCI from lowest Landau level behaviour. Our results are also of use in experimental design, as they provide a computationally inexpensive means to estimate which choices of FCI model parameters are most likely to yield a FQH-like state with the largest possible gap; a naive analysis of the scales involved has suggested this may be on the order of room temperature^8^. From the opposite point of view, our results also indicate which areas of parameter space should be searched to find possible FCI states which do not correspond to FQH universality classes.

## Results

### Geometry of Chern bands

We begin by introducing the quantities studied below. A necessary ingredient in engineering a fractional Chern insulator is a flat, topologically non-trivial band, defined as follows. Let 
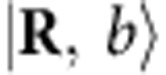
 be a tight-binding orbital localized at position **R**+**d**_*b*_; the Fourier transform of the *b*th basis orbital (where *b* ranges from 1 to 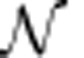) is


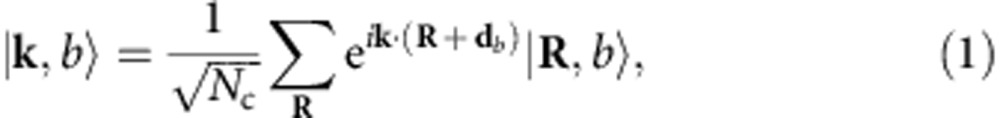


where **k** is a crystal momentum restricted to the first Brillouin zone (BZ) and *N*_c_ is the number of unit cells in the system, which are indexed by lattice vectors **R**. Eigenstates of the tight-binding Hamiltonian are Bloch functions


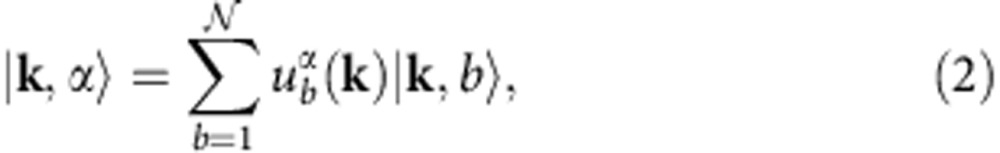


where *α* indices the bands. At a fixed **k**, the tight-binding Hamiltonian is an 
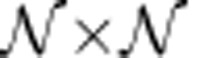
 matrix with entries


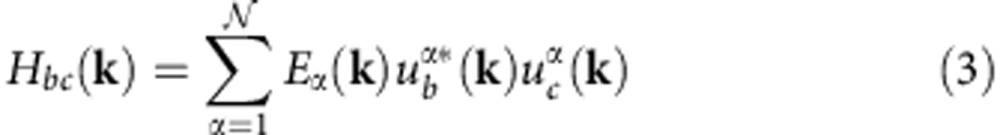


and band energies *E*_α_(**k**). In general, for 
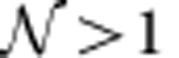
, neither *H*_*bc*_(**k**) nor 
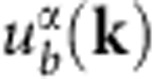
 will have the periodicity of the reciprocal lattice, and this property is not needed to define the Berry curvature via equation (5) below. Imposing this periodicity by hand has led to demonstrably incorrect calculations in previous literature. We clarify this point with a discussion in Supplementary Note 1 and illustrate the consequences of incorrect computations in Supplementary Figs 1,2.

Non-trivial topological order in a band α is indicated by a non-vanishing value of the (first) Chern number


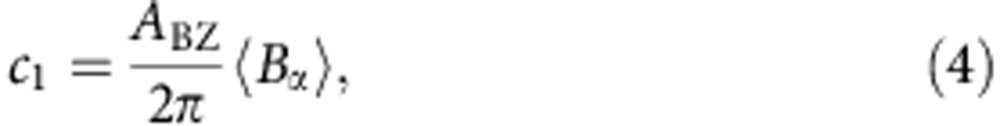


where *A*_BZ_ is the area of the momentum-space Brillouin zone, 
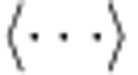
 denotes the average over the BZ, normalized so 
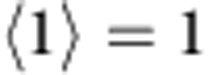
 and the Berry curvature^27^,^28^ of the band α is defined as


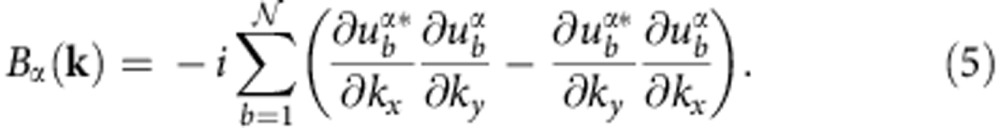


The leading-order condition (in terms of a long-wavelength expansion) for the existence of an isomorphism between the band-projected density operators and the GMP algebra found in ref. 26 is that the Berry curvature should be constant as a function of **k**. In the results we report here, we quantify fluctuations of Berry curvature over the BZ by their root-mean-square (RMS) value,


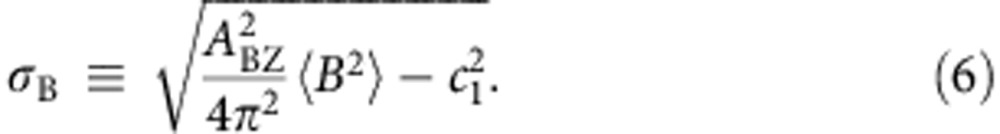


We normalize *σ*_B_ in the same way as the Chern number, so that (6) is dimensionless as well as insensitive to the scales over which deviations from the mean curvature occur.

The higher order conditions obtained ref. 26 involve the pull-back of the Fubini-Study metric on Hilbert space^29^, which we refer to below as the quantum metric. In terms of Bloch functions, it is given by


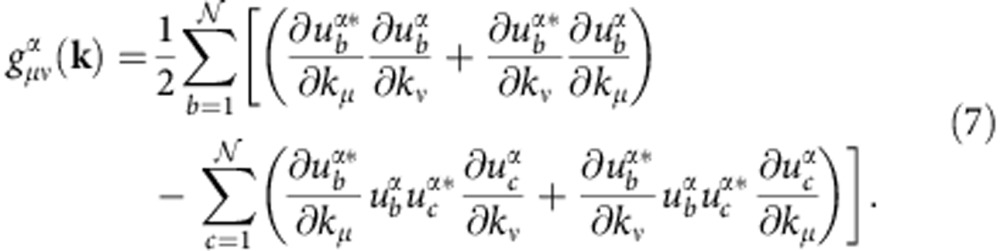


The next-to-leading-order condition of ref. 26 is that the quantum metric also be constant over the BZ. We adopt


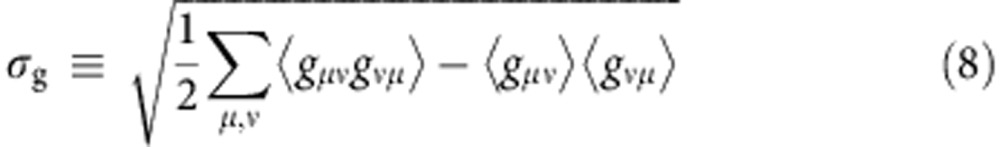


as the appropriate generalization of RMS fluctuation to tensor quantities. The final constraint on the band geometry is that


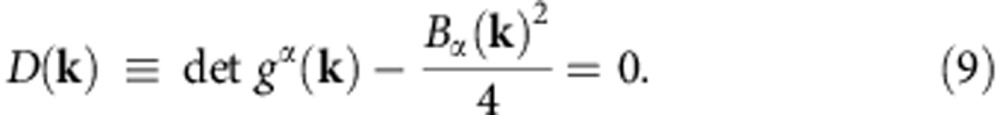


It was shown in ref. 26 that the left-hand side of (9) is always non-negative; the condition that it vanishes is equivalent to the condition that *g*^α^ and 
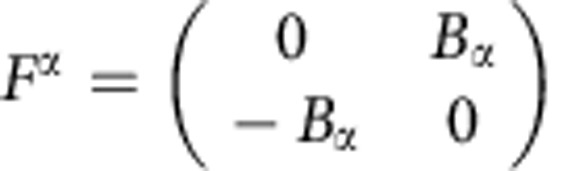
 are the real and imaginary components of a Kähler metric 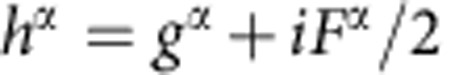. This means that, unlike the first two conditions, the metric determinant inequality *D*(**k**)≥0 measures deviations from lowest Landau level physics, specifically. Analogous conditions may be derived for higher Landau levels.

A stronger condition can be obtained by considering the trace of the quantum metric instead. It was additionally shown in ref. 26 that


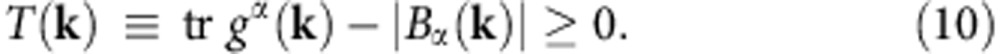


In Supplementary Note 2 we show that if this inequality is saturated, the quantum metric is isotropic and *D*(**k**) must vanish. Hence the condition *T*(**k**)=0 is equivalent to requiring that the algebra of band-projected density operators be identical to the GMP algebra, while *D*(**k**)=0 merely requires that they be isomorphic.

### Band geometry hypothesis

The purpose of the present work is to investigate the degree to which the above criteria are satisfied in several FCI models known to exhibit FQH-like phases^30^,^31^,^32^. In this section, we outline procedures common to all models studied.

The stability of an FCI phase is trivially influenced by the dispersion of the occupied band. Fortunately, the dispersion of a band is independent of its Berry curvature and quantum metric: the former only depends on the Hamiltonian's spectrum while the latter depend only on its eigenvectors. This allows us to eliminate any dispersion-related confounding effects by energetically flattening the bands of each lattice model, which is equivalent to smearing nearest-neighbour (NN) hopping terms over an exponentially localized area^9^,^11^.

Differences between Chern bands and Landau levels also enter in the form of the Hamiltonian's interaction term. Unlike the energetic considerations, this dependence is still poorly understood, so we have limited the scope of the present paper to on-site repulsive interactions only (which necessitates bosonic statistics), since this is the lattice interaction which most closely matches the isotropy present in the continuum. For each lattice model considered, we therefore investigate the bosonic Laughlin state at filling fraction *v*=1/2 (stabilized by a two-body delta-function interaction) and the bosonic Moore–Read state at *v*=1 (stabilized by a three-body delta-function interaction). These states have completely different topological orders; furthermore the Laughlin state is known to be more robust in general than the Moore–Read state, so examining both provides a useful probe of the sensitivity of band-geometric arguments.

The band geometry hypothesis predicts that the most important factor will be Berry curvature fluctuations. Low curvature fluctuations were heuristically identified as a desirable criterion in the earliest FCI literature^8^,^10^, which has been well established by subsequent work (see in particular refs 33, 34). In the present work we therefore focus on the sub-leading conditions, namely the influence the quantum metric has on the gap.

Fluctuations of the quantum metric are predicted to be the next most relevant quantity, but in the models examined this was found to have a high degree of linear correlation with the Berry curvature fluctuations (see Supplementary Fig. 3). There is no a priori reason this should be the case: other metric-derived quantities were found to be largely independent of Berry curvature. In addition, we found that the trace inequality (10) was far more correlated with the gap than the determinant inequality (9) for all models examined, despite corresponding to a stronger condition on the algebra of density operators. These findings go beyond the scheme laid out in ref. 26. Because of space constraints, we present data on the dependence of the gap on the determinant condition for low values of *σ*_B_ in Supplementary Fig. 4 for the kagomé lattice model and Supplementary Fig. 5 for the ruby lattice model.

### Haldane model

**Figure 1:**
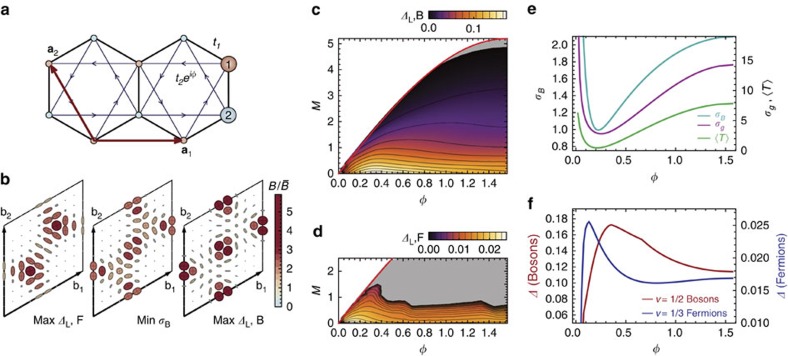
Band geometry and gap data for the Haldane model. (**a**) Honeycomb lattice used to define the Haldane model. Basis vectors **a**_1_, **a**_2_ are shown in red; basis elements are shown by differently coloured/numbered sites. Hopping elements are shown with black and blue edges; arrowheads indicate the chirality convention for complex hoppings. (**b**) Band geometry over the reciprocal lattice unit cell spanned by b_1_, b_2_, for parameter values maximizing the gap and minimizing *σ*_B_. Axes of ellipses are proportional to the eigenvectors of the quantum metric *g*^α^(k), and ellipse color is given by the relative deviation of Berry curvature *B*_α_(k) from its Brillouin zone-averaged value. (**c**) Gap *Δ* as a function of (*φ*, *M*) for *N*=8 bosons at *v*=1/2 with an on-site repulsion. (**d**) Gap as a function of (*φ*, *M*) for *N*=8 fermions at *v*=1/3 with nearest-neighbour repulsion. Note that this plot differs from Fig. 8 of ref. 32 because we exclude (light grey) parameters failing to meet energetic and entanglement-based criteria for Laughlin-type order. (**e**) Berry curvature fluctuations *σ*_B_ (left scale) and metric fluctuations *σ*_g_ and average trace inequality 
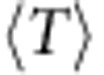
 (right scale) as functions of *φ* at *M*=0. (**f**) Reproduction of the gap data from **c**,**d** along *M*=0.

The first Chern insulator model was introduced by Haldane^35^, who considered a tight-binding model on the honeycomb lattice with nearest- and next-NN hoppings (Fig. 1a) and a Peierls phase due to non-uniform threading of magnetic flux through each hexagon. The single-particle Hamiltonian for the Haldane model is


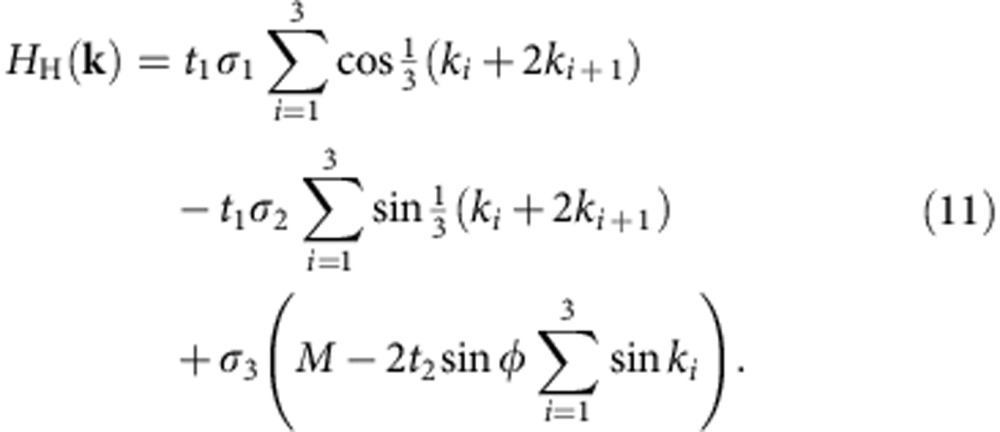


where the Pauli sigma matrices act on the band index, *k*_*i*_≡**k**·**a**_*i*_, **a**_3_=−**a**_1_–**a**_2_ and *i*=1,2,3 is interpreted cyclically mod 3. The lower band has a non-zero Chern number when 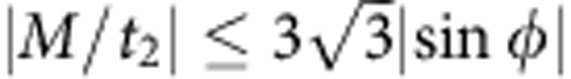. This model has been extensively studied numerically, and the addition of short-ranged repulsive interactions has been shown to yield both the bosonic^30^,^31^ and fermionic^32^ Laughlin states at appropriate filling fractions.

For purposes of comparison with refs 32, 33, we consider the model at *t*_1_=*t*_2_=1. The energy spectrum has band crossings for these parameters, meaning that the bands cannot be flattened by local operators and the model analysed in those references is not adiabatically connected to (11). In practice, however, one is most interested in the *M*=0 subspace; the Hamiltonian then depends only on the combination *t*_2_ sin *φ*/*t*_1_, and an increase in *t*_2_/*t*_1_ which removes the crossing may then be compensated by a shift in *φ* which leaves the Hamiltonian (11) unchanged up to a scale.

The momentum dependence of the Berry curvature and quantum metric is shown in Fig. 1b for parameters which minimize *σ*_B_ and maximize the gaps for the Laughlin state of bosons and fermions; these values are listed in Supplementary Table 1. We see that the distribution of Berry curvature minimizing *σ*_B_ interpolates between that which maximizes the gap for the bosonic and fermionic Laughlin states and likewise the value of *φ* minimizing *σ*_B_ lies between the values minimizing the gaps (Fig. 1e,f). The band geometry argument does not distinguish the statistics of the underlying particles, because of the fact that the projected density operators are bilinear in particle operators and bosonic in either case. The (*φ*, *M*) parameter space may be sampled exhaustively; many-body gaps for the bosonic and fermionic Laughlin states are shown over the full topologically non-trivial region of parameter space in Fig. 1c,d. The largest gaps and most uniform band geometry both occur for *M*=0.

The other band-geometric criteria are highly correlated with the curvature fluctuation *σ*_B_ and yield little new information for this model (Fig. 1e), beyond being close to the location of the maximum gaps (Fig. 1f). We prove in Supplementary Note 3 that the remaining band-geometric criterion, the determinant condition (9), is necessarily saturated for any two-band model, but the trace condition (10) remains non-trivial here.

### Augmented Haldane model

Although the Haldane model at fractional filling exhibits a robust Laughlin state, its Berry curvature remains highly non-uniform even in the best case (Fig. 1b). We would like to be able to compare this model with the kagomé and ruby lattice models, in which more uniform curvature may be achieved. In addition, since we are interested in the sub-leading effects the quantum metric has on the gap, we want to examine band configurations for which the metric is more independent of the Berry curvature than in Fig. 1e.

This may be accomplished by adding a third-NN hopping term, with independent coupling *t*_3_, to the Haldane model Hamiltonian (11). In the previous section, we saw that a sub-lattice chemical potential *M* always reduces the gap of an FCI phase, so we set *M*=0 below. The remaining couplings in the new Hamiltonian may be parameterized by 
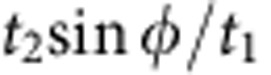
 and *t*_3_/*t*_1_, so the interesting region of parameter space is still two-dimensional.

**Figure 2:**
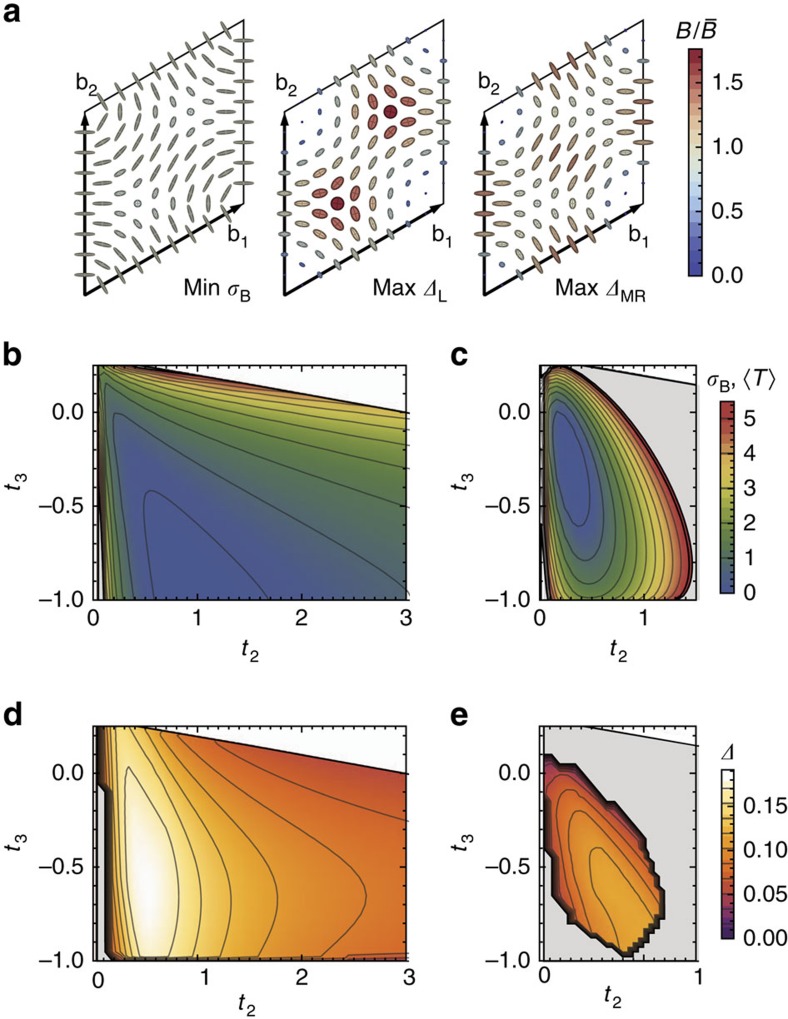
Band geometry and gap data for the augmented Haldane model. (**a**) Band geometry over the reciprocal lattice unit cell spanned by b_1_, b_2_, for parameter values maximizing the gaps and minimizing *σ*_B_. Axes of ellipses are proportional to the eigenvectors of the quantum metric *g*^α^(k). Ellipse colour is given by the relative deviation of Berry curvature *B*_α_(k) from its Brillouin zone-averaged value. (**b**) Berry curvature fluctuations *σ*_B_ and (**c**) Brillouin zone-averaged trace inequality 
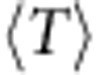
 as a function of couplings *t*_2_, *t*_3_, where we set *t*_1_=1 and *φ*=*π*/2 in this and remaining panels without loss of generality. (**d**) Gap *Δ* as a function of couplings for the bosonic Laughlin state of *N*=8 bosons at *v*=1/2. (**e**) Gap as a function of couplings for the bosonic Moore–Read state of *N*=10 bosons at *v*=1.

The momentum dependence of the Berry curvature and quantum metric is shown in Fig. 2a for parameters which minimize *σ*_B_ and maximize the gaps for the Laughlin and Moore–Read states; these values are given in Supplementary Table 2. Comparison with Fig. 1b shows that curvature fluctuations have been reduced; furthermore, the minimum of *σ*_B_ (shown in Fig. 2b) occurs at different parameter values than the minimum of the trace condition (shown in Fig. 2c). Gaps for the Laughlin and Moore–Read states are shown in Fig. 2d,e, respectively; the maximum gaps in both cases occur at lower values of *t*_2_ than the minimum value of *σ*_B_, which one may attribute to the influence of 
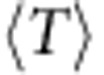
.

### Kagomé lattice model

**Figure 3:**
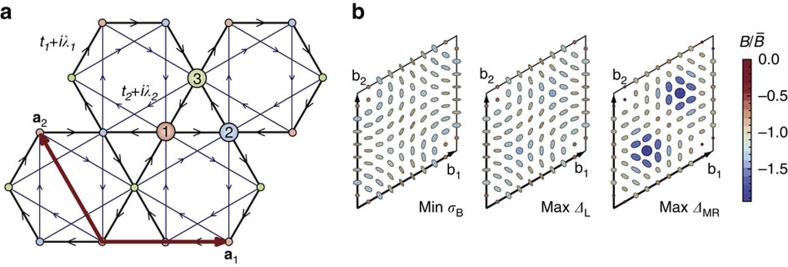
Definition of couplings and band geometry for the kagomé lattice model. (**a**) Lattice and chirality conventions for hopping terms in the kagomé lattice model Hamiltonian. Basis vectors **a**_1_, **a**_2_ are shown in red; basis elements are shown by differently coloured/numbered sites. Hopping elements are shown with black and blue edges; arrowheads indicate the chirality convention for complex hoppings. (**b**) Band geometry over the reciprocal lattice unit cell spanned by b_1_, b_2_, for parameter values minimizing *σ*_B_ and maximizing gaps, respectively. Axes of ellipses are proportional to the eigenvectors of the quantum metric *g*^α^(k). Ellipse color is given by the relative deviation of Berry curvature *B*_α_(k) from its Brillouin zone-averaged value.

A Chern insulator defined on the kagomé lattice was introduced by Tang *et al.*^8^ (Fig. 3a). This model is attractive for our purposes since it has three bands, while remaining structurally similar to the Haldane model.

Defining a complex hopping matrix element for the relative embedding of the sublattices in the unit cell as


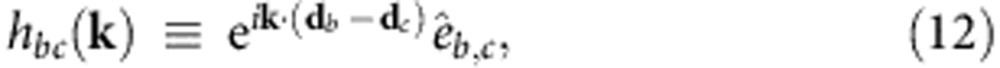


where 
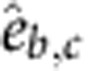
 is the unit matrix whose (*b*, *c*)th entry is equal to 1, the momentum-space Hamiltonian for the kagomé lattice model is


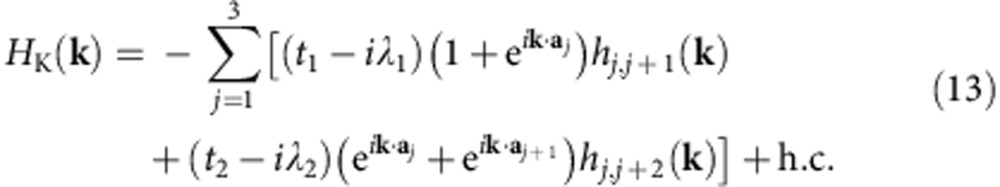


where h.c. is an abbreviation for the hermitian conjugate, **a**_3_=−**a**_1_−**a**_2_, and *j* is interpreted cyclically mod 3. The relative offsets **d**_*b*_ are as depicted by the numbered sites in Fig. 3a. The momentum dependence of the Berry curvature and quantum metric is shown in Fig. 3b for parameters minimizing *σ*_B_ and maximizing the gaps for the Laughlin and Moore–Read states; these values are listed in Supplementary Table 3.

**Figure 4:**
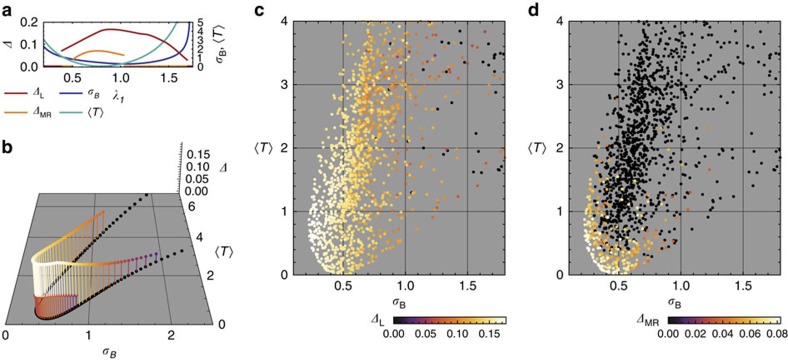
Gap versus band geometry for the kagomé lattice model. (**a**) Gap and geometry data for the kagomé lattice model with NN-only hoppings, as a function of the only coupling ratio λ_1_/*t*_1_. (**b**) The same data as a one-dimensional submanifold in band geometry space, which we parameterize in terms of Berry curvature fluctuations *σ*_B_ and the averaged trace condition 
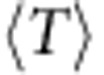
. The upper set (larger gap *Δ*) of points are gaps for the *N*=8 bosonic Laughlin state at *v*=1/2, while the lower are for the bosonic Moore–Read state at *v*=1. (**c**) Gap for the kagomé lattice model with both nearest-neighbour and next-nearest-neighbour couplings, as a function of band-geometric parameters (*σ*_B_, 
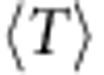
) for the Laughlin state of *N*=8 bosons at *v*=1/2. (**d**) Gap as a function of (*σ*_B_, 
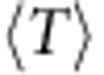
) for the Moore–Read state of *N*=10 bosons at *v*=1, for the same coupling values.

We first consider the model with NN hoppings only (Fig. 4a,b). Because band geometry is determined by single-particle quantities, the values of 
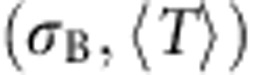
 for a given value of the effective NN coupling λ_1_/*t*_1_ are identical for the Laughlin and Moore–Read states. Despite having different topological orders, gaps for both states decline monotonically as one proceeds from the region of minimum *σ*_B_ and 
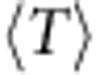
, and this trend holds over the entire phase (that is, up to the closure of the gap).

To establish that the above trends were not coincidental, we studied the entire *c*_1_=−1 phase containing the NN-only point *t*_1_=λ_1_; *t*_2_=λ_2_=0 for the bosonic Laughlin (Fig. 4c) and Moore–Read (Fig. 4d) states. The gap's sensitivity to band geometry is most apparent for the more fragile Moore–Read state: the state is only stable in a small region, with the largest gaps (white points) attained at parameters with the lowest values of *σ*_B_ and 
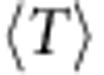
 found. The fact that the region of stability is an arc, rather than a vertical line, demonstrates that 
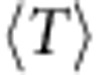
 describes independent, non-negligible factors influencing the stability of this state. These phenomena are less evident in the Laughlin state (Fig. 4c), which remains stable over a wide range of parameter values.

**Figure 5:**
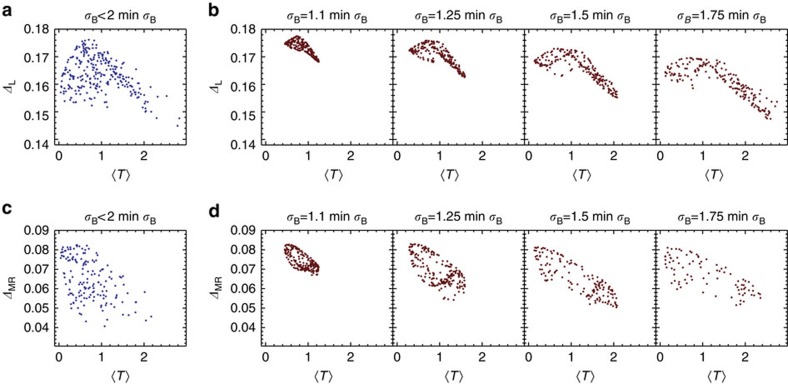
Gap versus trace condition for the kagomé lattice model subject to constraints on *σ*_B_. (**a**) Gaps for the bosonic Laughlin state of *N*=8 bosons at *v*=1/2, as a function of the Brillouin zone average of the trace condition 
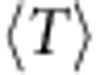
. We only plot gaps for parameter values which have Berry curvature fluctuations *σ*_B_ less than twice its minimum value. (**b**) Gap of the bosonic Laughlin state versus 
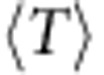
 for parameter values randomly chosen on isosurfaces of constant *σ*_B_ in the space of couplings. The parameter space sampling procedure used to obtain these sets of points is described in the Methods. (**c**,**d**) The same, for the bosonic Moore–Read state of *N*=10 bosons at *v*=1. Note that the same sets of model parameters are used in each column.

Because quantum metric-dependent quantities enter at a higher order than Berry curvature fluctuations in the band geometry analysis, they should have a subdominant effect on the gap. In Fig. 5 we control for the effects of large curvature fluctuations by restricting attention to parameter values giving near-minimal values of *σ*_B_. Including all such parameters yields a one-way relationship for the Laughlin and Moore–Read states (Fig. 5a,c), in the sense that large gaps are obtained only at low values of 
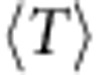
, but small gaps can be obtained at any value of 
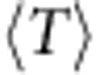
. Further detail is evident if we take parameter values chosen to give the same value of *σ*_B_ (see the Methods for a description of the sampling procedure used.) Results for sets of points chosen to have four different values of *σ*_B_ are shown in Fig. 5b,d for the Laughlin and Moore–Read states. Removing the variation in *σ*_B_ reveals a full-fledged negative correlation between 
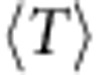
 and the gaps: the trend is approximately linear for models having curvature fluctuations near the minimum and becomes less so as curvature fluctuations are allowed to increase.

### Ruby lattice model

**Figure 6:**
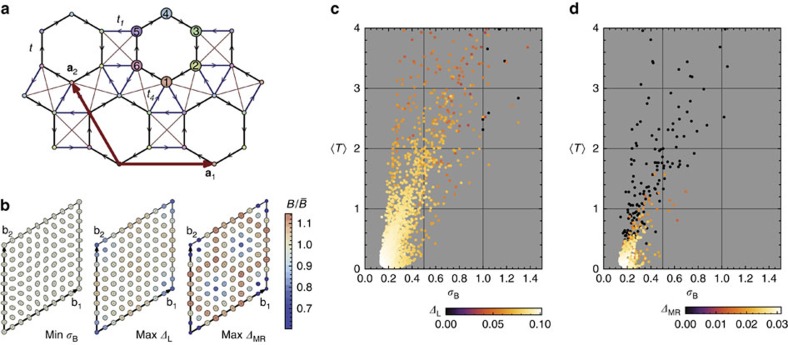
Definition of couplings, band geometry and gaps for the ruby lattice model. (**a**) Lattice and chirality conventions for hopping terms in the ruby lattice model Hamiltonian. All nearest-neighbour (arrowed) bonds are the same length. Basis vectors **a**_1_, **a**_2_ are shown in red; basis elements are shown by differently coloured/numbered sites. Hopping elements are shown with black (*t*), blue (*t*_2_) and brown (*t*_4_) edges; arrowheads indicate the chirality convention for complex hoppings. (**b**) Band geometry over the reciprocal lattice unit cell spanned by b_1_, b_2_, for parameter values minimizing *σ*_B_ and maximizing gaps, respectively. Axes of ellipses are proportional to the eigenvectors of the quantum metric *g*^α^(k). Ellipse color is given by the relative deviation of Berry curvature *B*_α_(k) from its Brillouin zone-averaged value. (**c**) Gap *Δ* as a function of the average Berry curvature fluctuation *σ*_B_ and averaged trace condition 
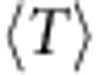
 for the Laughlin state of *N*=8 bosons at *v*=1/2. (**d**) Gap as a function of (*σ*_B_, 
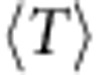
) for the Moore–Read state of *N*=10 bosons at *v*=1.

Hu *et al.*^36^ described a Chern insulator model on the ruby lattice (Fig. 6a). In the limit of total spin polarization, they showed that hopping parameters could be chosen such that the lowest band had *c*_1_=1 and a bandgap to bandwidth ratio of ∼70. The Hamiltonian for this model is


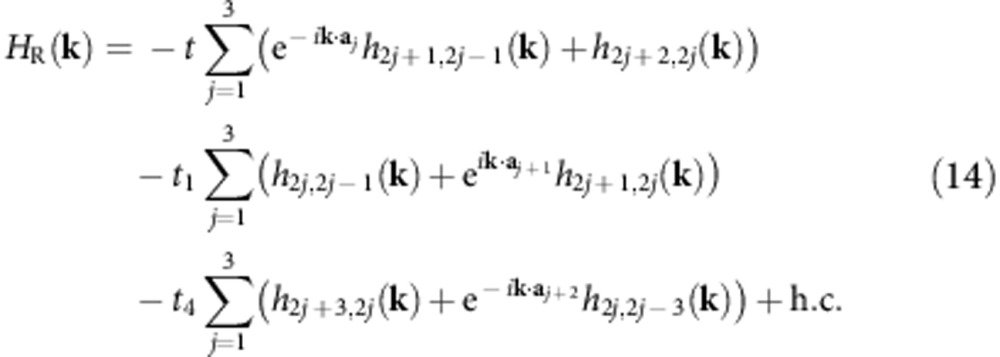


where *h*_*b,c*_(**k**) is defined in equation (12). Here **a**_3_=−**a**_1_−**a**_2_ and the index on **a** is interpreted cyclically mod 3, but the indices on *h*_*b,c*_(**k**) are interpreted cyclically mod 6. The relative offsets **d**_*b*_ are as depicted by the numbered sites in Fig. 6a. We considered the *c*_1_=1 phase containing the flat-band point found in ref. 36, with *t*=1.0+1.2*i*, *t*_1_=−1.2+2.6*i*, and *t*_4_=−1.2. Momentum dependence of the Berry curvature and quantum metric is shown in Fig. 6b for parameters which minimize *σ*_B_ and maximize the gaps for the Laughlin and Moore–Read states; these values are listed in Supplementary Table 4. Remarkably, the complexity of this Hamiltonian works in our favour: one can find parameter values which greatly reduce the fluctuations in band geometry relative to the kagomé lattice model, which means that the ruby lattice model may be tuned to produce a much closer approximation to lowest Landau level physics.

**Figure 7:**
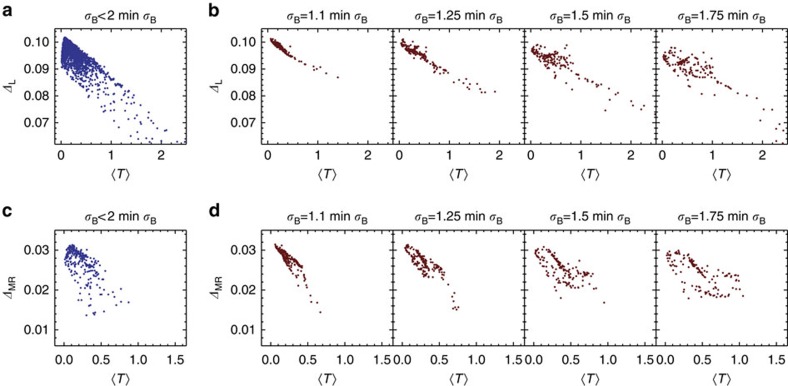
Gap versus trace condition for the ruby lattice model subject to constraints on *σ*_B_. (**a**) Gaps for the bosonic Laughlin state of *N*=8 bosons at *v*=1/2, as a function of the Brillouin zone average of the trace condition 
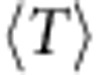
. We only plot gaps for parameter values which have Berry curvature fluctuations *σ*_B_ less than twice its minimum value. (**b**) Gap of the bosonic Laughlin state versus 
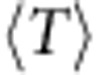
 for parameter values randomly chosen on isosurfaces of constant *σ*_B_ in the space of couplings. (**c**,**d**) The same, for the bosonic Moore–Read state of *N*=10 bosons at *v*=1. Note that the same sets of model parameters are used in each column.

As a consequence, trends identified in the kagomé lattice model are manifest here with a much higher degree of correlation. Fig. 6c,d show that the gaps of the Laughlin and Moore–Read states are strongly correlated with band geometry as measured by both *σ*_B_ and 
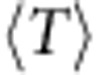
. In both cases the gap can be seen to decrease with increasing 
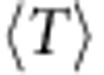
, even for the same values of *σ*_B_; in particular, the Moore–Read state is only stable in the lower right half of the plot area. Restricting our attention to parameters yielding small fluctuations in Berry curvature, in Fig. 7 we display data analogous to that presented for the kagomé lattice model in Fig. 5. Again, if we only impose an upper bound on *σ*_B_ we see that it's not possible to have a large gap for large values of 
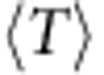
 (Fig. 7a,c), while upon restricting to specific values of *σ*_B_ (Fig. 7b,d) we see that the negative correlation between 
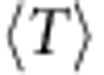
 and the gaps is even tighter, becoming nearly linear for low *σ*_B_ and gaining more scatter as *σ*_B_ is increased.

### Significance of correlations

In this section we describe two approaches to quantifying the degree of correlation between the band geometry and the many-body gaps described above. In particular, the fact that FQH-type states are destabilized by fluctuations in Berry curvature is readily apparent and was anticipated in the first work on FCIs; in the present work, we are interested in possible additional dependence on conditions derived from the quantum metric, so we seek to measure correlation between the gap and the trace condition 
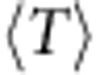
. This is not fully straightforward, because of correlation of 
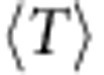
 and *σ*_B_ with each other, as evidenced by the fact that parameter values are not uniformly distributed in Figs 4c and 6c.

One approach is to compare data for parameters yielding the same value of *σ*_B_, some of which is shown in Figs 5b,d and 7b,d (see the Methods for the procedure used to sample from isosurfaces of constant *σ*_B_). We do not have quantitative predictions for the functional dependence of the gap on any band-geometric quantity, so to avoid introducing assumptions we use Spearman's *ρ* as a nonparametric measure of correlation. This is defined as the linear (Pearson) correlation coefficient between the rankings of the data points when rank-ordered by *Δ* and by 
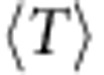
, and takes values ranging from +1 for any monotonically increasing function to −1 for any monotonically decreasing function.

**Figure 8:**
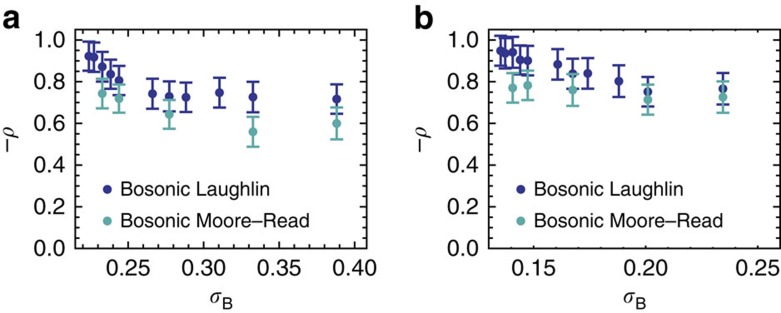
Correlation between gaps and the trace condition when the magnitude of Berry curvature fluctuations is held constant. Each data point shows (the negative of) Spearman's *ρ* for the correlation between gap and averaged trace condition 
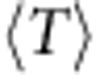
 on separate samples of *n*=200 points sampled from isosurfaces of constant root-mean-square Berry curvature fluctuation *σ*_B_. A value of *ρ*=−1 corresponds to perfectly monotonic anti-correlation. We show results for Laughlin (dark blue) and Moore–Read (light blue) states in (**a**) the kagomé lattice model and (**b**) the ruby lattice model. Error bars show the bootstrapped s.e.m.

In Fig. 8 we plot the results of this test for the Laughlin and Moore–Read data in the kagomé and ruby lattice models. This score was found to be within ∼10% of the linear correlation between 
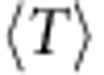
 and *Δ* for each isosurface, which indicates the robustness of our conclusion and implies that all values of *σ*_B_ considered lie in a weak-fluctuation regime. The general trend evident in Fig. 8 is that *Δ* is highly negatively correlated with 
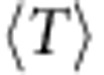
 when curvature fluctuations are constrained to be near their minimum value. As curvature fluctuations are allowed to increase, this relationship becomes less exact, but converges to an asymptotic value well above 0 for both the Laughlin and Moore–Read states. This confirms the qualitative picture evident in Figs 5b,d and 7b,d.

Alternatively, we can analyze the larger set of data having unrestricted values of *σ*_B_, at the cost of assuming linear relationships between all variables; the similarity between the results for Spearman's and Pearson's *ρ* mentioned above suggest that this is justified. One can then compute the partial correlation, denoted here by *ρ*_B_, as the degree of linear correlation between the residuals of *Δ* and 
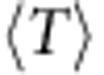
, after first subtracting the best-fit linear dependence of each on *σ*_B_; similarly, this score ranges from +1 to −1. For the augmented Haldane, kagomé and ruby lattice models, respectively, we find *ρ*_B_=−0.91, −0.42 and −0.62 for the Laughlin state and *ρ*_B_=−0.77, −0.44 and −0.46 for the Moore–Read state. Sample sizes were, respectively, *n*=1,900, 2,100 and 1,800. The fact that these values are lower than those obtained by the isosurface method describes the non-negligible correlation between *σ*_B_ and 
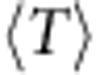
.

All correlation scores quoted above and shown in Fig. 8 are statistically significant at the 1% level (at most). This is the main result of this section: gaps for both states are independently sensitive to variations in the trace condition, beyond the correlations induced in the latter via curvature fluctuations. This confirms that band geometry plays a significant role in realistic FCI models.

### Cross-model comparisons

The band geometry hypothesis claims that the most stable FQH-like phases are obtained when the band geometry is tuned to be as close to that of a Landau level as possible. We have shown above that this holds for several different lattice models as their couplings are varied, but comparison of the models shows an apparent contradiction: the gaps reported above are smallest for the ruby lattice model, despite the fact that this model can be made to approximate Landau level physics more closely than the other models studied here.

The resolution of this apparent contradiction lies in the fact that the models considered have different numbers of tight-binding sites per unit cell. This factor enters into the interaction term of the Hamiltonian, and hence the gap: because we have flattened the dispersion of the kinetic term, the interaction strength is the only energy scale in the problem. In Supplementary Note 4 we give a scaling argument that the strength of a delta-function interaction in the continuum should be multiplied by a factor of 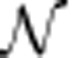 when discretized to an on-site repulsion in a model with 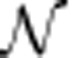 bands. This means that, when comparing the gaps of bosonic Laughlin states in different models, the appropriate quantity to compare is 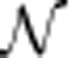
*Δ*. For the three-body delta-function interaction which stabilizes the bosonic Moore–Read state, the same considerations yield a scaling factor of 
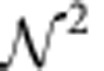
.

**Figure 9:**
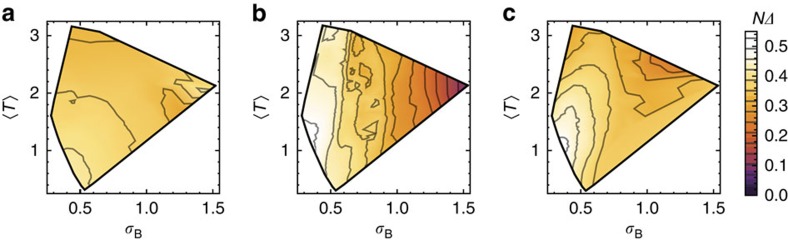
Cross-model comparison of scaled gaps as a function of band geometry. Panels show interpolated energy gaps of the bosonic Laughlin state, scaled by the number of bands of each model, for the (**a**) augmented Haldane, (**b**) kagomé lattice and (**c**) ruby lattice models described above. Scaled gaps are plotted as a function of band geometry as measured by Berry curvature fluctuations *σ*_B_ and the average value of the trace condition 
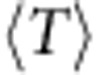
. Data are only plotted in the region of common overlap of the three models in (*σ*_B_, 
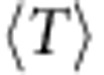
) space (polygonal outline). The colour scale and contours used are identical for all three panels.

In Fig. 9 we compare the gaps for the Laughlin state for the augmented Haldane, kagomé and ruby lattice models when scaled by this factor, as a function of the band-geometric parameters (*σ*_B_, 
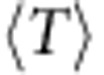
). The domain of each plot is restricted to be the common overlap of the three models in (*σ*_B_, 
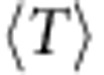
) space. Because of this restriction, we were unable to perform a meaningful analysis for the Moore–Read state, as it is unstable in most of this region (compare Figs 4d and 6d). Despite the fact that the scaling argument is exact only in the large-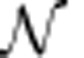 limit and the models we compare have 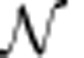
=2,3 and 6, we find roughly similar behaviour across all three models, both in terms of the magnitude of the scaled gap and of its dependence on *σ*_B_ and 
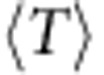
.

## Discussion

In this work we have presented numerical results which systematically map out the robustness of topologically ordered FQH-like phases in FCI systems with realistic, short-ranged Hamiltonians and band geometry which is less than perfectly uniform. We presented quantitative evidence that the band-geometric quantities identified in ref. 26 remain strongly correlated with the size of the gap even when its conditions on band geometry are not met exactly. This leads us to propose a geometric stability hypothesis for FQH-like phenomena in FCIs: in spite of the fact that the GMP algebra is not perfectly reproduced, we conjecture that an approximate version of the single-mode approximation correctly describes the low-energy physics of these FCI models. As a practical corollary, we predict that single-particle Hamiltonians with more uniform band geometry—specifically, as measured by the hierarchy of three criteria—will produce more stable FQH-like states.

The validity of the single-mode approximation in FCIs has been investigated by a number of other authors using approaches complementary to that taken here^25^,^26^,^33^,^37^. Within the context of their Hamiltonian approach to the FQHE, Murthy and Shankar showed that composite fermion degrees of freedom could be chosen which reproduce an exact version of the GMP algebra^38^,^39^. More directly, in ref. 40, the lowest-lying neutral excitation of the kagomé and ruby lattice models was found to be well described by the magnetoroton mode of the corresponding FQH state on a torus, using a phenomenological mapping between the FQH and FCI Hilbert spaces described in ref. 41. A related mapping was originally proposed by Qi^42^, but the image of FQH pseudopotential interactions under this mapping is not well localized and strongly anisotropic^42^,^43^,^44^,^45^,^46^, making the relationship to physical FCI Hamiltonians unclear.

Comparing the results from the kagomé and ruby lattice models, it appears easier to engineer uniform geometry in more complicated Hamiltonians, both in the sense of having more tunable couplings and in the sense of having more bands. The latter property is expected to hold on general grounds, as noted in refs 13, 47. Increasing the size of the unit cell reduces the effectiveness of a fixed-strength repulsion, however, so an optimal choice would balance these two factors. This has immediate relevance to experimental design: laboratory Hamiltonians are necessarily more complicated than those in idealized theoretical models (for example, the proposal in ref. 16 involves an eight-dimensional parameterization of the applied electric field used to obtain a synthetic gauge potential.) Performing many-body simulations on a representative set of parameters in such a large space is prohibitively time-consuming; the geometric stability hypothesis can be used to reduce this to a manageable subspace. In addition, band geometry may, by definition, be tuned independently of energetic considerations such as the bandwidth.

A pressing direction for future work is to further develop the band geometry hypothesis by investigating its validity in less straightforward scenarios: stable FCI states where the Berry curvature is not particularly uniform have also been proposed; furthermore, the stability of the state may also depend on the filling fraction and the particular state sought to be stabilized^48^,^49^. Among other aspects which would be interesting to clarify are the role of bosonic versus fermionic particle statistics, NN- and longer-ranged inter-particle interactions, anisotropic interactions induced by the lattice structure and so on. In particular, the distribution of geometric quantities differs from band to band; this can be selected by fully filling a number of bands in a fermionic system, which could lead to new phenomena. One could also consider the wide range of more elaborate FCI models in the literature, possessing, for example, Chern numbers 
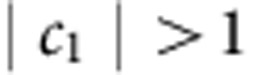
, non-Abelian Berry curvature arising from multiple degenerate bands. We direct the reader to the recent reviews^50^,^51^ for a more extensive discussion and bibliography.

## Methods

### Parameter space sampling

We study the dependence of the many-body gap on band-geometric quantities in the Haldane model^35^ and models proposed for the kagomé^8^ and ruby^36^ lattices. The parameter space for the Haldane model Hamiltonian is small enough that the vector of parameters *t*_Δ_ which maximize the gap *Δ* may be found from exhaustive sampling. The parameter spaces for the other two models are higher-dimensional and non-compact, so this strategy will not work.

According to the geometric stability hypothesis, the Berry curvature fluctuations *σ*_B_ should be inversely correlated with the gap, meaning that it may be employed as a proxy for the latter which requires far less effort to compute. In Supplementary Note 5 we derive an expression for the derivative of *σ*_B_ with respect to any parameter appearing in the single-particle Hamiltonian, allowing steepest-descent methods to be employed. Of course, we do not expect the vector of parameters *t*_0_ minimizing *σ*_B_ to precisely coincide with those maximizing the gap, but if the single-mode approximation is applicable, *t*_0_ will be a viable initial guess for *t*_Δ_, to be refined as described below. Indeed, a state with a robust topological gap in the presence of large curvature fluctuations would be of immediate interest as an FCI phase not describable in terms of an FQH universality class.

**Figure 10:**
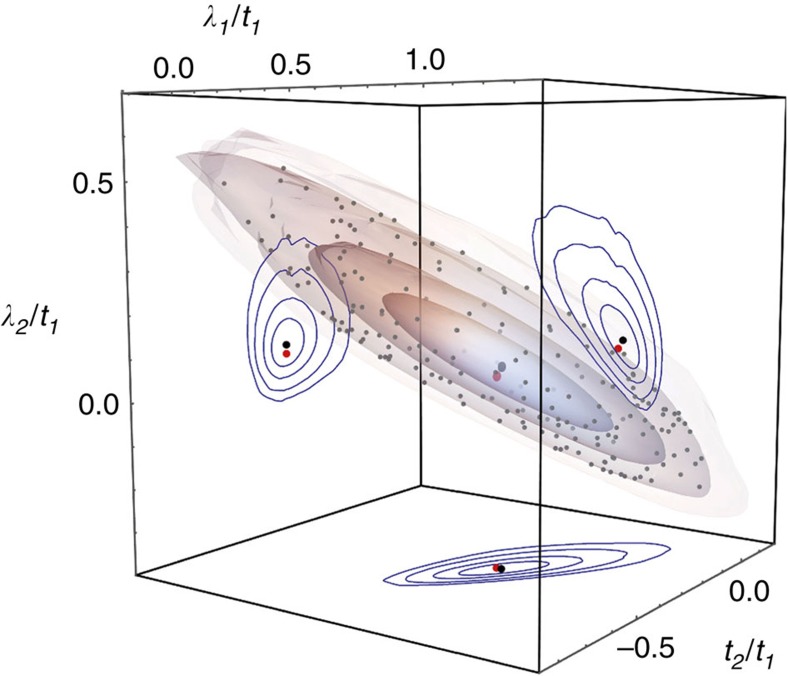
Isosurfaces of constant Berry curvature fluctuations in the parameter space of the kagomé lattice model. Parameter values which minimize the root-mean-square Berry curvature fluctuation *σ*_B_ of the kagomé lattice model are marked by the large, central black dot. The large red dot identifies parameter values which yielded the maximum gap for the Laughlin state of *N*=8 bosons at *v*=1/2. Concentric shaded surfaces are isosurfaces upon which *σ*_B_ takes a constant value equal to 1.05, 1.1, 1.25 and 1.5 times its minimum value (respectively). Blue contours on the walls of the box are sections through these surfaces at the minimum *σ*_B_ point. Small grey points identify random samples taken from the *σ*_B_=1.25 min *σ*_B_ isosurface, to illustrate uniformity.

In the neighbourhood of *t*_0_, a surface of constant *σ*_B_ in parameter space will be approximated by an ellipsoid given by the Hessian of *σ*_B_(*t*) at *t*_0_, which may be calculated by the method in Supplementary Note 5. We sample points uniformly from the surface of the ellipsoid defined by the Hessian by the well-known method of projecting vectors of parameters *t* sampled from the corresponding multi-normal distribution. We then perturb this ellipsoid by shifting the vectors *t* radially along the rays connecting each with *t*_0_ until we find parameters *t*′ such that *σ*_B_(*t*′) is equal to the target value. These isosurfaces of different parameters with the same value of *σ*_B_ permit us to study the sub-leading effects of the quantum metric on the gap predicted by the geometric stability hypothesis: in Fig. 10 we depict the isosurfaces found for the kagomé lattice model which were used to generate the data shown in Fig. 5b,d.

Finally, we locate the parameters giving the maximum gap by fitting a quadratic form


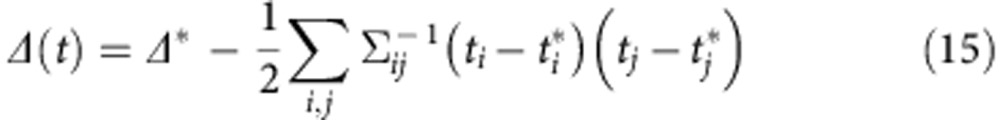


to the gaps from the isosurface data and sampling new parameters from a Gaussian distribution centred on t* with covariance matrix Σ. We have verified that deviations of the actual *t*_Δ_ found from this data from the fitted value t* are negligible compared to the scales set by Σ, hence this search does not need to be iterated further.

### Numerical exact diagonalization

Unless explicitly identified otherwise, all data were obtained from exact diagonalization of the many-body Hamiltonian for *N*=8 bosons interacting with a two-body on-site repulsion at a filling fraction (ratio of the number of particles to the number of unit cells) of *v*=1/2 on a periodic lattice of 4 × 4 unit cells (for the Laughlin-like FCI state) or *N*=10 bosons at a filling fraction of *v*=1 on a lattice of 5 × 2 unit cells, interacting with a three-body on-site repulsion only (for the Moore–Read-like FCI state). In the main text we also presented additional data obtained for the fermionic Laughlin-like state in the Haldane model with *N*=8 particles at v=1/3 on a 6 × 4 lattice with two-body nearest-neighbour repulsion. The fact that the bosonic Moore–Read state is realized in a filled Landau level means that, relative to the Laughlin state, we are able to simulate more particles with a many-body Hilbert space of roughly the same size. Flattening the spectrum of the single-particle Hamiltonian removes the only other energy scale from the problem, so the strength of the repulsive interaction sets the units of the many-body gap *Δ*.

Because we do simulations for thousands of parameter values, a detailed finite-size scaling analysis of each is beyond the scope of this paper. We have, however, made spot checks by doing simulations of *N*=6–12 particles at select parameter values. Representative data is shown in Supplementary Fig. 6. From this analysis, we have concluded that the system sizes described above strike an acceptable balance between accurately approximating the thermodynamic (*N*→∞) limit and making the volume of simulations manageable on the computational resources available to us.

We employ several criteria to identify topological order in the results of numerical simulations, employing information from both the many-body energy spectrum and the many-body wavefunctions themselves. We first require that the spectrum has the correct number of degenerate ground states in the proper momentum sectors (two states at (*k*_*x*_, *k*_*y*_)=(0, 0) for bosonic Laughlin-type order, and three states at (*k*_*x*_, *k*_*y*_)=(0, 0), (*k*_*x*_, *k*_*y*_)=(0, 0) and (0, 1) for bosonic Moore–Read-type order). In a finite-size simulation these ground states will only be approximately degenerate; we require that the gap to the lowest-energy excited state (out of all momentum sectors; that is, the indirect gap) be at least as large as the spread in ground state energies.

We ascertain whether a ground state wavefunction has the topological order of the Laughlin or Moore–Read state through properties of its entanglement spectrum^52^: we first verify that the spectrum of the reduced density matrix obtained by tracing out four bosons is gapped. There is no current quantitative theoretical interpretation of the magnitude of the entanglement gap; instead, the discriminative power of this criterion comes from requiring that the number of eigenvalues below the gap in each momentum sector obeys counting rules dictated by the topological order of continuum FQH state^11^,^41^. For the lattice geometry used in our studies, Laughlin-type order in a system of *N*=8 bosons at *v*=1/2 is identified by the (1, 2) counting rule (in the terminology of the previous references), which requires 48 states below the gap at (*k*_*x*_, *k*_*y*_)=(0, 0), 44 states below the gap in sectors (*k*_*x*_, *k*_*y*_)=(0, 2), (0, 2) and (2, 2) and 40 states below the gap in all other momentum sectors. Similarly, the counting rule for Moore–Read-type order in *N*=10 bosons at *v*=1 requires 76 states below the gap at (*k*_*x*_, *k*_*y*_)=(0, 0) and (0, 1) and 75 states below the gap in all other momentum sectors. Simulations that fail any of the above tests are assigned an energy gap of zero.

## Additional information

**How to cite this article:** Jackson, T. S. *et al.* Geometric stability of topological lattice phases. *Nat. Commun.* 6:8629 doi: 10.1038/ncomms9629 (2015).

## Supplementary Material

Supplementary Information Supplementary Figures 1-6, Supplementary Tables 1-4, Supplementary Notes 1-5 and Supplementary References.
