# Supplementary material for: Geometric stability of topological lattice phases
Source: Nat Commun. 2015 Nov 4;6:8629. doi: 10.1038/ncomms9629 (PMC4659836; doi:10.1038/ncomms9629)
Supplement: Supplementary Information — Supplementary Figures 1-6, Supplementary Tables 1-4, Supplementary Notes 1-5 and Supplementary References. [file ncomms9629-s1.pdf]

Supplementary Information for "Geometric stability of topological lattice phases"

T. S. Jackson, Gunnar Möller, and Rahul Roy

(Dated: July 15, 2015)

SUPPLEMENTARY FIGURES

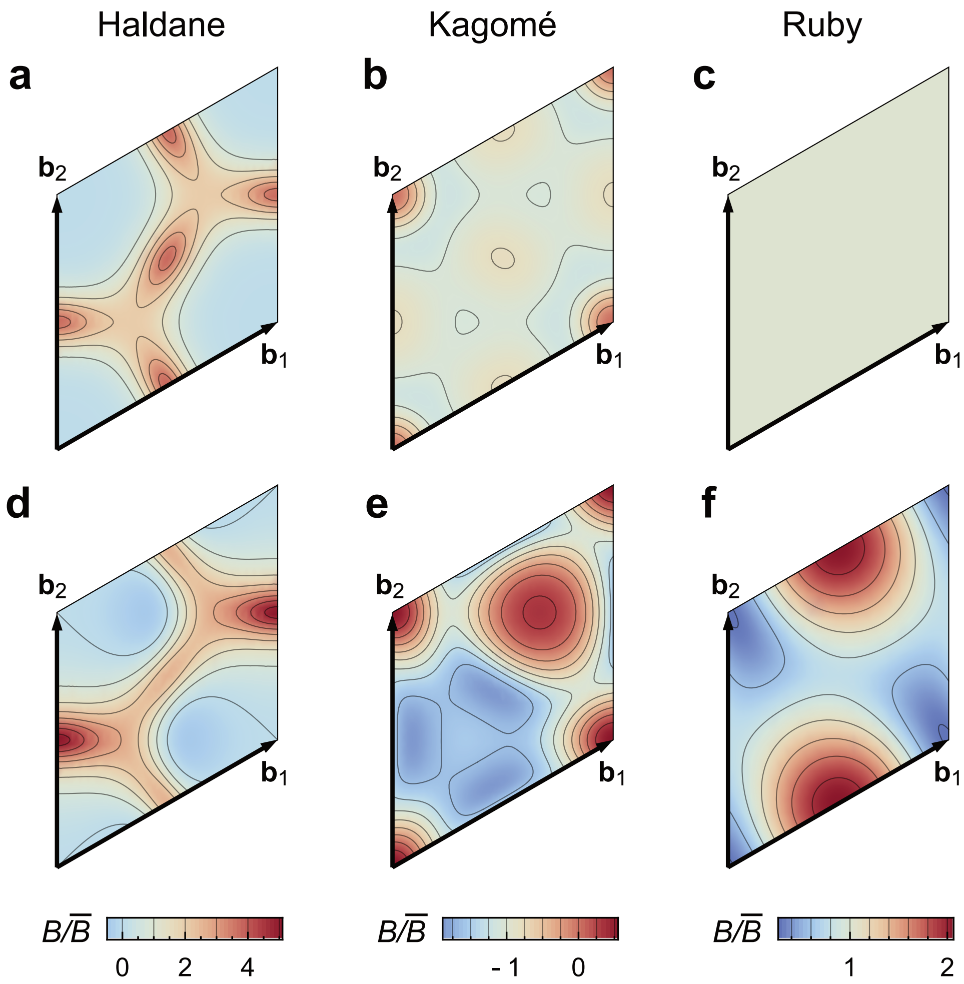

Supplementary Figure 1 | Effect of Hamiltonian transformation on momentum space distribution of Berry curvature. We plot the deviations from uniform Berry curvature (neutral grey color) over a unit cell of the reciprocal lattice spanned by $\mathbf{b}_1, \mathbf{b}_2$, for models treated in the main text. Plots in each column use the same color scale and set of contours, as indicated. (**a**) – (**c**) Berry curvature distribution for the Haldane, kagomé lattice and ruby lattice models, respectively, at parameter values which minimize the root-mean-square curvature fluctuation σ_B over the unit cell. Curvature fluctuations of the ruby lattice model at its $\min \sigma_B$ point (**c**) are too small to be visible on the common scale. (**d**) – (**f**) Berry curvature distribution for the transformed versions of these models used in Supplementary Ref. 1, evaluated the parameter values minimizing the values of σ_B as computed with the transformed Hamiltonians.

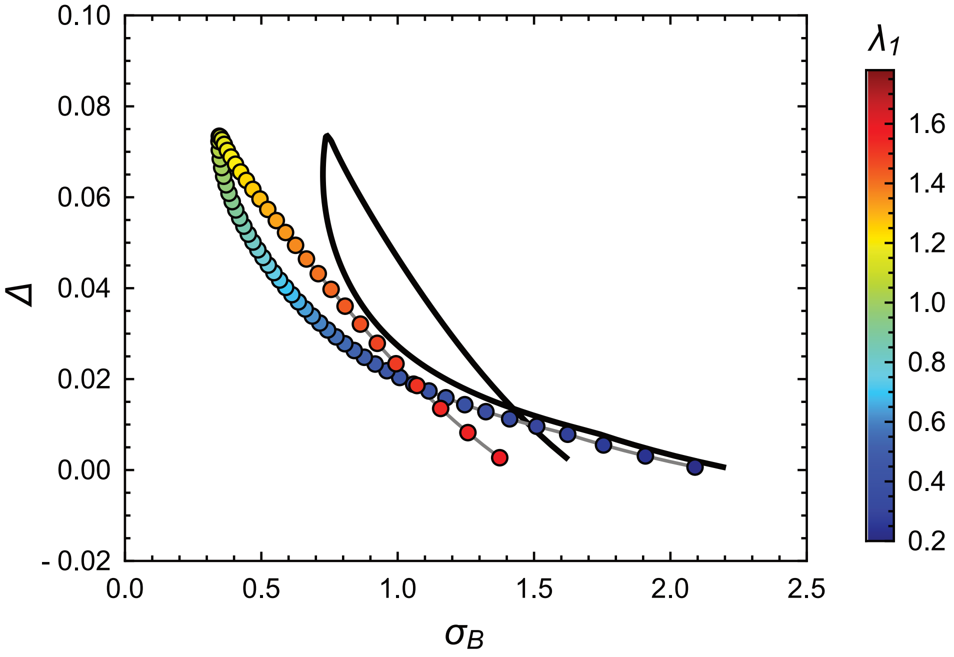

Supplementary Figure 2 | Effect of Hamiltonian transformation on Berry curvature-gap correlations. Representative plot of the correlation between the many-body gap Δ and root-mean-square Berry curvature fluctuation σ_B. Data shown are for the Laughlin state of $N = 8$ fermions in the kagomé lattice model with nearest-neighbor repulsion on a 6×4 lattice. The single-particle parameters used are $t_2 = \lambda_2 = 0$ and $t_1 = 1$, with the color scale corresponding to λ_1. This model and parameters are chosen to match those used in Supplementary Ref. 1 (compare Fig. 26 of that reference); the thick black line corresponds to values of σ_B computed using the transformed Hamiltonian of that reference, while colored circles denote values of σ_B computed using the Hamiltonian in the main text. The two sets of data have the same gaps; only the computed values of σ_B differ.

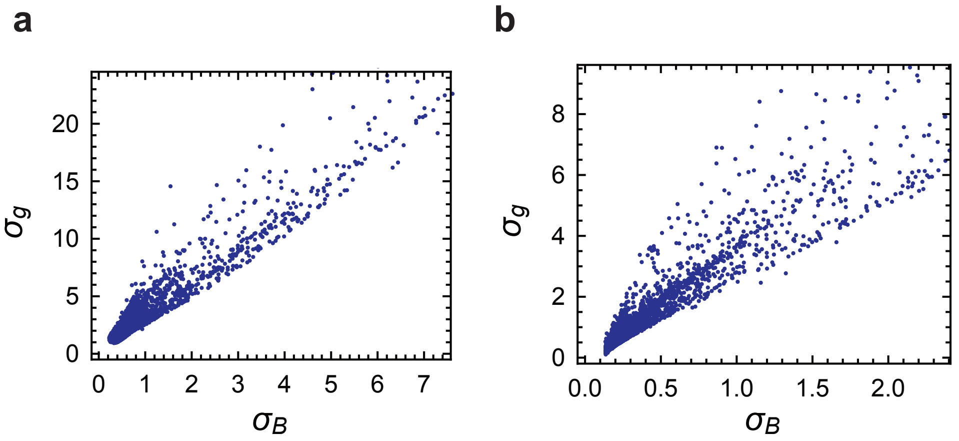

Supplementary Figure 3 | Correlation between fluctuations of Berry curvature and of the quantum metric. The root-mean-square (RMS) fluctuation of the quantum metric over the Brillouin zone (σ_g) is plotted against the RMS fluctuation of the Berry curvature σ_B, for all parameter values used in the text for the (**a**) the kagomé lattice model and (**b**) the ruby lattice model. Despite sampling a large volume of the parameter space of both models, the fluctuations in both quantities show a large degree of linear correlation.

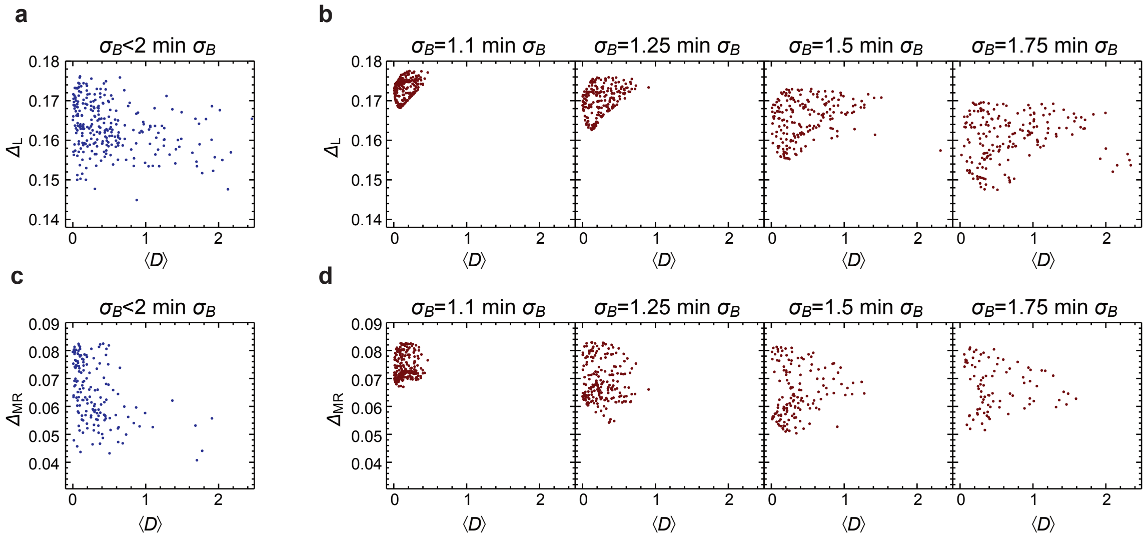

Supplementary Figure 4 | Gap vs. determinant condition for the kagomé lattice model subject to constraints on σ_B. (**a**) Gaps for the bosonic Laughlin state of $N = 8$ bosons at $\nu = 1/2$, as a function of the Brillouin zone average of the determinant condition $\langle D \rangle$. We only plot gaps for parameter values which have Berry curvature fluctuations σ_B less than twice its minimum value. (**b**) Gap of the bosonic Laughlin state vs. $\langle D \rangle$ for coupling values randomly chosen on isosurfaces of constant σ_B in the space of couplings. The parameter space sampling procedure used to obtain these sets of points is described in the main text (see Methods). (**c**), (**d**) The same, for the bosonic Moore-Read state of $N = 10$ bosons at $\nu = 1$. Note that the same sets of couplings are used in each column.

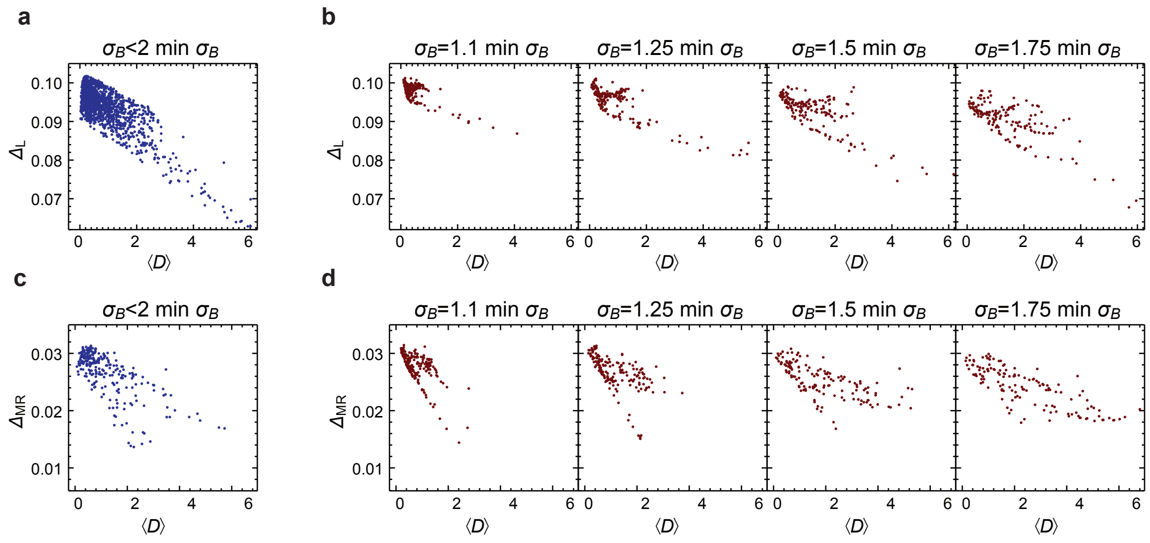

Supplementary Figure 5 | Gap vs. determinant condition for the ruby lattice model subject to constraints on σ_B. (**a**) Gaps for the bosonic Laughlin state of $N = 8$ bosons at $\nu = 1/2$, as a function of the Brillouin zone average of the determinant condition $\langle D \rangle$. We only plot gaps for parameter values which have Berry curvature fluctuations σ_B less than twice its minimum value. (**b**) Gap of the bosonic Laughlin state vs. $\langle D \rangle$ for coupling values randomly chosen on isosurfaces of constant σ_B in the space of couplings. The parameter space sampling procedure used to obtain these sets of points is described in the main text (see Methods). (**c**), (**d**) The same, for the bosonic Moore-Read state of $N = 10$ bosons at $\nu = 1$. Note that the same sets of couplings are used in each column.

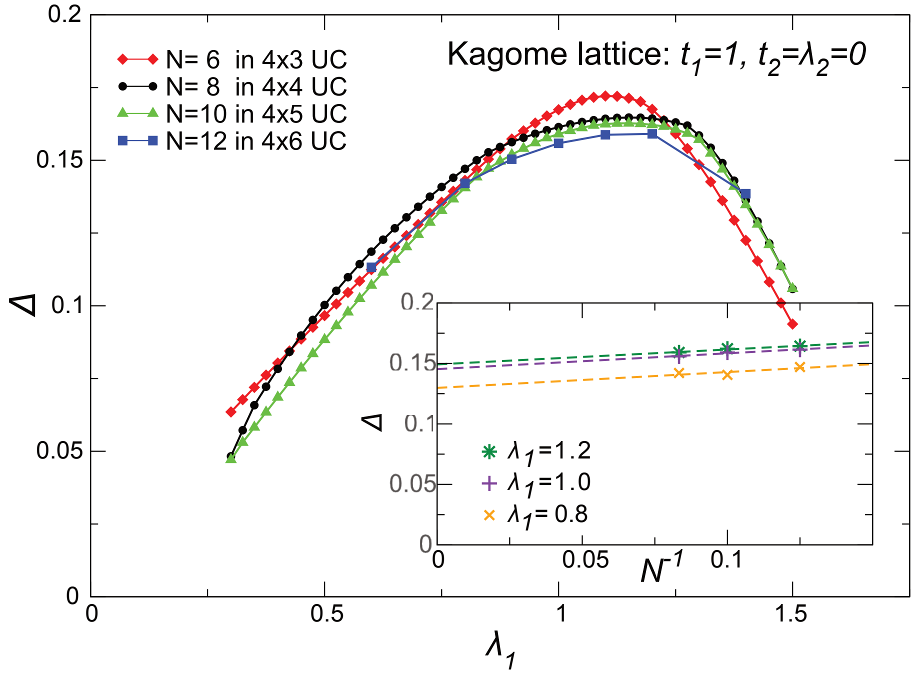

Supplementary Figure 6 | Finite-size scaling of the many-body gap in the kagomé lattice model with nearest-neighbor hoppings. The many-body gap data presented in the main text were obtained for systems of $N = 8$ particles, which represents a compromise between the need to accurately estimate the gap in thermodynamic limit and the need to exhaustively sample the parameter space of single-particle Hamiltonians using the computational resources available to us. Here we plot the many-body gap Δ for the bosonic Laughlin state in the kagomé lattice model with nearest-neighbor hopping $t_1 = 1$, variable λ_1, and next-nearest-neighbor hopping $t_2 = \lambda_2 = 0$. Red, black, green and blue points denote data for systems with $N = 6$, 8, 10 and 12 particles, respectively: the behavior changes significantly between system sizes $N = 6$ and $N = 8$, but the gap data for $N = 10$ and $N = 12$ closely mirrors that of $N = 8$, especially near the maximum of the gap. Inset: finite size scaling of the gap as a function of $1/N$ for selected values of λ_1. Near the maximal gap, the thermodynamic extrapolation ($N \to \infty$) for the gap differs from the finite size value at $N = 8$ by about 10%.

SUPPLEMENTARY TABLES

Supplementary Table 1 | Parameter values, band-geometric quantities and gaps for the Haldane model.

	$\min \sigma_B$	$\max \Delta_{\mathrm{L}}$, Fermions	$\max \Delta_{\mathrm{L}}$, Bosons
ϕ	0.225	0.131	0.354
M	0.0	0.0	0.0
σ_B	0.99326	1.24755	1.12760
σ_g	2.85734	3.97585	3.25586
$\langle D \rangle$	0	0	0
$\langle T \rangle$	0.46705	0.84597	0.99358
Δ		0.02554	0.17243

As in the main text, we take the hopping amplitudes to be $t_1 = t_2 = 1$, with the remaining couplings ϕ, M as free variables. Columns list information for the following values of (ϕ, M) of interest: those which minimize Berry curvature fluctuations σ_B (obtained using the steepest-descent procedure described in Supplementary Note 5) and those maximizing the many-body gap Δ for the Laughlin state for $N = 8$ fermions at $\nu = 1/3$ and $N = 8$ bosons at $\nu = 1/2$. The latter two parameter values were found using our parameter space sampling procedure (see Methods). For these parameters, we compute the root-mean-square average over the Brillouin zone of fluctuations in the Berry curvature (σ_B) and quantum metric (σ_g), as well as the Brillouin zone averages of the determinant ($\langle D \rangle$) and trace ($\langle T \rangle$) conditions.

Supplementary Table 2 | Parameter values, band-geometric quantities and gaps for the augmented Haldane model with third-nearest neighbor hopping.

	$\min \sigma_B$	$\max \Delta_{\mathrm{L}}$	$\max \Delta_{\mathrm{MR}}$
t_2	1.0	0.55	0.75
t_3	-1.0	-0.65	-0.65
σ_B	0.14699	0.43579	0.34909
σ_g	3.00134	1.70829	2.29776
$\langle D \rangle$	0	0	0
$\langle T \rangle$	3.44154	0.75821	1.46273
Δ		0.19153	0.12614

As in the main text, we fix $t_1 = 1$, $\phi = \pi/2$, and $M = 0$, allowing t_2, t_3 to vary. Columns list information for the following values of (t_2, t_3) of interest: those which minimize Berry curvature fluctuations σ_B (obtained using the steepest-descent procedure described in Supplementary Note 5) and those maximizing the many-body gap Δ for the Laughlin state of $N = 8$ bosons at $\nu = 1/2$ and for the Moore-Read state of of $N = 10$ bosons at $\nu = 1$. The latter two parameter values were found using our parameter space sampling procedure (see Methods). For these parameters, we compute the root-mean-square average over the Brillouin zone of fluctuations in the Berry curvature (σ_B) and quantum metric (σ_g), as well as the Brillouin zone averages of the determinant ($\langle D \rangle$) and trace ($\langle T \rangle$) conditions.

Supplementary Table 3 | Parameter values, band-geometric quantities and gaps for the kagomé lattice model.

	$\min \sigma_B$	$\max \Delta_{\mathrm{L}}$	$\max \Delta_{\mathrm{MR}}$
λ_1	0.745	0.725	0.934
t_2	−0.361	−0.361	−0.168
λ_2	0.078	0.055	−0.129
σ_B	0.22178	0.23231	0.34967
σ_g	1.31746	1.25972	1.25434
$\langle D\rangle$	0.09608	0.09857	0.41665
$\langle T\rangle$	0.79612	0.70181	0.42712
Δ		0.17614	0.08246

We fix an energy scale by setting $t_1 = 1$ and letting the remaining model parameters (λ_1, t_2, λ_2) vary. Columns list information for the following parameter values of interest: those which minimize Berry curvature fluctuations σ_B (obtained using the steepest-descent procedure described in Supplementary Note 5) and those maximizing the many-body gap Δ for the Laughlin state of $N = 8$ bosons at $\nu = 1/2$ and for the Moore-Read state of of $N = 10$ bosons at $\nu = 1$. The latter two parameter values were found using our parameter space sampling procedure (see Methods). For these parameters, we compute the root-mean-square average over the Brillouin zone of fluctuations in the Berry curvature (σ_B) and quantum metric (σ_g), as well as the Brillouin zone averages of the determinant ($\langle D\rangle$) and trace ($\langle T\rangle$) conditions.

Supplementary Table 4 | Parameter values, band-geometric quantities and gaps for the ruby lattice model.

	$\min \sigma_B$	$\max \Delta_{\mathrm{L}}$	$\max \Delta_{\mathrm{MR}}$
Im t	1.857	1.886	1.672
Re t_1	0.685	−0.569	−1.261
Im t_1	2.079	2.536	2.789
t_4	−2.118	−2.022	−2.007
σ_B	0.13398	0.14347	0.17980
σ_g	0.47669	0.21574	0.38617
$\langle D\rangle$	0.17740	0.25569	0.30456
$\langle T\rangle$	0.17168	0.08262	0.11139
Δ		0.10173	0.03132

We fix an energy scale by setting Re $t = 1$ and letting the remaining parameters (Im t, t_1, t_4) vary. Columns list information for the following parameter values of interest: those which minimize Berry curvature fluctuations σ_B (obtained using the steepest-descent procedure described in Supplementary Note 5) and those maximizing the many-body gap Δ for the Laughlin state of $N = 8$ bosons at $\nu = 1/2$ and for the Moore-Read state of of $N = 10$ bosons at $\nu = 1$. The latter two parameter values were found using our parameter space sampling procedure (see Methods). For these parameters, we compute the root-mean-square average over the Brillouin zone of fluctuations in the Berry curvature (σ_B) and quantum metric (σ_g), as well as the Brillouin zone averages of the determinant ($\langle D\rangle$) and trace ($\langle T\rangle$) conditions.

SUPPLEMENTARY NOTES

Supplementary Note 1: Gauge freedom and geometric phases in band Hamiltonians

Here we discuss issues arising in the definition of Berry curvature for Bloch bands which are absent from the general formalism of geometric phases. The issues identified here are not new;[2,3] we discuss them here in order to demonstrate that the values the Berry curvature and quantum metric take at a specific crystal momentum $\mathbf{k}$ are unambiguously defined and, in principle, measurable.

Berry curvature arises in physical applications at the as the commutator of band-projected position operators; as examples, we cite the semiclassical approximation to the orbital magnetization[4] which contributes to anomalous thermoelectric transport, and the intrinsic contribution to the anomalous Hall conductance[5]

$$\sigma_{xy}^{\text{int}} = \frac{e^2}{\hbar} \int \frac{d^d k}{(2\pi)^d} f(E_{\mathbf{k}}) B(\mathbf{k}), \tag{1}$$

Other applications are reviewed in Supplementary Ref. 6. We mention these observables here because they depend on the Berry curvature through forms *other* than its Brillouin zone (BZ) average (the Chern number), meaning that the distribution of curvature within the BZ is an experimentally meaningful quantity. There is also a recent experimental proposal to measure the Berry curvature directly.[7]

Textbook discussions of Berry's phase are usually framed in the context of adiabatic evolution of a quantum state tracing out a closed cycle in some parameter manifold. In Chern insulator applications, the parameter manifold is the Brillouin zone, and the instantaneous eigenfunctions at a parameter $\mathbf{k}$ are the spatially periodic part of the Bloch functions $u_b^\alpha(\mathbf{k})$. The Berry connection is $\mathbf{A}_\alpha(\mathbf{k}) = -i\langle u_{\mathbf{k}}^\alpha | \nabla_{\mathbf{k}} | u_{\mathbf{k}}^\alpha \rangle$, which has curvature given by

$$B_\alpha(\mathbf{k}) = -i \sum_{b=1}^{\mathcal{N}} \left(\frac{\partial u_b^{\alpha *}}{\partial k_x} \frac{\partial u_b^\alpha}{\partial k_y} - \frac{\partial u_b^{\alpha *}}{\partial k_y} \frac{\partial u_b^\alpha}{\partial k_x} \right). \tag{2}$$

The first Chern number is defined as the surface integral of the Berry curvature

$$c_1 = \frac{1}{2\pi} \int_{BZ} d^2k \, B_\alpha(\mathbf{k}) \tag{3}$$

and is topologically quantized to integer values due to the fact that the Brillouin zone is a compact manifold (a torus).

Eigenstates of the band Hamiltonian

$$H_{bc}(\mathbf{k}) = \sum_{\alpha=1}^{\mathcal{N}} E_\alpha(\mathbf{k}) u_b^{\alpha*}(\mathbf{k}) u_c^\alpha(\mathbf{k}) \tag{4}$$

are only defined up to an overall phase

$$|u_{\mathbf{k}}^\alpha\rangle \to e^{i\phi_\alpha(\mathbf{k})}|u_{\mathbf{k}}^\alpha\rangle, \tag{5}$$

where $\phi_\alpha(\mathbf{k})$ is any smooth function satisfying $\phi_\alpha(\mathbf{k}+\mathbf{G}) = \phi_\alpha(\mathbf{k})$. This is the gauge symmetry of the band Hamiltonian, and it is the only such symmetry in the absence of energy degeneracies (assumed for simplicity throughout this section). As with the $U(1)$ gauge symmetry of electromagnetism, gauge transformations of the form (5) alter the Berry connection but leave the Berry curvature (analogue of the magnetic field) and quantum metric unchanged, as can be seen from their explicitly gauge-invariant forms (28), (29).

The reader will note that the band Hamiltonians used in this article do *not* have the periodicity of the reciprocal lattice; nor do their eigenfunctions $|u_{\mathbf{k}}^\alpha\rangle$, and so neither can immediately be viewed as functions on the BZ torus defined by identifying the points $\mathbf{k}$ and $\mathbf{k}+\mathbf{G}$ for any reciprocal lattice vector $\mathbf{G}$. We first explain that the formalism of band geometry is unchanged in this situation, and then argue that this choice of basis is the canonically correct one, in the sense of corresponding to the observable quantities mentioned above.

Because the full Hamiltonian is periodic in real space, Bloch's theorem implies

$$u_{\mathbf{k}}^\alpha(\mathbf{r}=\mathbf{d}_b) \equiv \langle \mathbf{0}b|u_{\mathbf{k}}^\alpha\rangle = \sum_{\mathbf{G}} c_{\mathbf{k}-\mathbf{G}} e^{i\mathbf{G}\cdot\mathbf{d}_b} \tag{6}$$

is unchanged under $\mathbf{d}_b \to \mathbf{d}_b + \mathbf{R}$; in other words, there exists a unitary matrix $U_{\mathbf{G}} = e^{i\mathbf{G}\cdot\hat{\mathbf{r}}}$ such that

$$u_b^\alpha(\mathbf{k}+\mathbf{G}) = \sum_{c=1}^{\mathcal{N}} (U_{\mathbf{G}})_{bc} u_c^\alpha(\mathbf{k}), \tag{7}$$

for *all* $\mathbf{k}, \alpha$. Because $U_{\mathbf{G}}$ is independent of $\mathbf{k}$, it drops out of the expressions for the Berry curvature and quantum metric, which are therefore periodic in $\mathbf{k}$.

One could also obtain manifestly periodic Bloch functions by performing momentum-dependent phase shifts $c_{b,\mathbf{k}}^\dagger \to e^{-i\mathbf{r}_b\cdot\mathbf{k}} c_{b,\mathbf{k}}^\dagger$, with different offsets $\mathbf{r}_b$ for each sublattice b, so that the transformed Bloch functions are invariant under $\mathbf{k} \to \mathbf{k}+\mathbf{G}$: the transformed curvature and metric are then periodic as well.

The resulting Hamiltonian is, of course, gauge-inequivalent to the original one, which can be seen from the fact that the curvature itself changes: under transformations

$$u_a^\alpha(\mathbf{k}) \to \widetilde{u}_b^\alpha(\mathbf{k}) = e^{i\mathbf{r}_b\cdot\mathbf{k}} u_b^\alpha(\mathbf{k}) \tag{8}$$

for $b = 1, \ldots, \mathcal{N}$, the Berry curvature at $\mathbf{k}$ changes by

$$\widetilde{B}_\alpha(\mathbf{k}) - B_\alpha(\mathbf{k}) = \sum_{b=1}^{\mathcal{N}} r_{b,y} \frac{\partial}{\partial k_x} |u_b^\alpha(\mathbf{k})|^2 - r_{b,x} \frac{\partial}{\partial k_y} |u_b^\alpha(\mathbf{k})|^2. \tag{9}$$

Because this is a sum of total derivatives, the surface integrals of $\widetilde{B}_\alpha(\mathbf{k})$ and $B_\alpha(\mathbf{k})$ yield the same Chern number. The difference itself, however, only vanishes when $\mathbf{r}_b$ is the same for all b, which is the gauge transformation (5).

Phase shifts of the form (8) were employed in recent publications[1,8] to obtain band Hamiltonians that were periodic in $\mathbf{k}$. This was described as a "gauge transformation" in these references, but as we've noted, the only gauge symmetry of the Hamiltonian is with respect to *bands* (i.e. $U(1)$ rotations in the eigenbasis $\gamma_{\mathbf{k}}^{\alpha\dagger} \to e^{-i\phi_\alpha(\mathbf{k})}\gamma_{\mathbf{k}}^{\alpha\dagger}$). For the transformations made in Supplementary Ref. 1, the difference in curvature fluctuations is substantial, as shown in Supplementary Fig. 1 for the Haldane, kagomé lattice and ruby lattice models. Note that each panel of this figure shows the curvature distribution for parameters which minimize σ_B as computed with each panel's respective Hamiltonian; i.e. they depict the closest one may get to uniform band geometry in the parameter space of the Hamiltonian considered.

For completeness, we note that unlike these single-particle properties, the many-body gap is invariant under the generalized phase shift (8), because single-particle density operators $\overline{\rho}_{\mathbf{k}}$ are left unchanged by the transformation. As an example, in Supplementary Fig. 2 we reproduce the results shown in Fig. 26 of Supplementary Ref. 1, along with the fluctuations in the canonically defined Berry curvature for the same system. The latter are lower than in the transformed Hamiltonian used in that reference, meaning that the negative correlation between σ_B and the gap is stronger than depicted there: introducing phase shifts by hand distorts the Berry curvature distribution to a degree which significantly affects the conclusions one may draw from that data.

We have shown that band geometry may be defined for non-periodic band Hamiltonians; we now argue that the non-periodic basis used in this paper is in fact the one measured by any (direct or indirect) experimental probe, and hence is the only one which should be regarded as physical. The feature possessed by Bloch bands which is absent from the general theory of Berry phases is the fact that the parameter space in question is defined via the Fourier transform of the kinematic setting of the physical system. Because

this is a *global* transform, in doing the Fourier sum

$$|\mathbf{k}, b\rangle = \frac{1}{\sqrt{N_c}} \sum_{\mathbf{R}} e^{i\mathbf{k}\cdot(\mathbf{R}+\mathbf{d}_b)} |\mathbf{R}, b\rangle, \tag{10}$$

we have already implicitly chosen a basis for the band Hilbert space at each $\mathbf{k}$. For example, the the transformations made in Supplementary Refs. 1 and 8 correspond to defining modified tight-binding states on the reciprocal lattice via

$$\widetilde{\chi}_b(\mathbf{k}) = \frac{1}{\sqrt{N_c}} \sum_{\mathbf{R}} e^{i\mathbf{k}\cdot\mathbf{R}} \chi_b(\mathbf{r} - \mathbf{R} - \mathbf{d}_b) \tag{11}$$

where $\chi_b(\mathbf{r} - (\mathbf{R} + \mathbf{d}_b)) = \langle \mathbf{r} | \mathbf{R} b \rangle$; this is manifestly invariant under $\mathbf{k} \to \mathbf{k} + \mathbf{G}$, but in doing so the position operator is no longer consistently defined for orbitals with different offsets $\mathbf{d}_b$: the transformation is equivalent to shifting the orbitals of the crystal basis to the origins of various lattice cells. As an obviously apparent consequence, the crystal symmetry of the transformed Berry curvatures in the second row of Supplementary Fig. 1 are unphysically broken. Because experiments probe position-space quantities, we conclude that the basis defined by (10) and used in this paper is the canonical, physically relevant quantity.

Supplementary Note 2: Relation between the trace and determinant inequalities

The inequalities

$$\text{tr}\, g^\alpha(\mathbf{k}) \geq |B_\alpha(\mathbf{k})|; \tag{12}$$

$$\det g^\alpha(\mathbf{k}) \geq B_\alpha(\mathbf{k})^2/4, \tag{13}$$

were proved in Supplementary Ref. 9. Assuming the quantum metric is nondegenerate, it may be factored as

$$g^\alpha = \sqrt{g^\alpha} \begin{pmatrix} \tilde{g}_{11} & \tilde{g}_{12} \\ \tilde{g}_{12} & \tilde{g}_{22} \end{pmatrix} \tag{14}$$

where the scalar $\sqrt{g^\alpha} = (\det g^\alpha)^{1/2}$ and the second factor is a unimodular matrix. Using the standard inequality between arithmetic and geometric means,

$$\text{tr}\, g^\alpha = \sqrt{g^\alpha}(\tilde{g}_{11} + \tilde{g}_{22}) \geq 2\sqrt{g^\alpha}\sqrt{\tilde{g}_{11}\tilde{g}_{22}}. \tag{15}$$

By unimodularity $\sqrt{\tilde{g}_{11}\tilde{g}_{22}} = \sqrt{1+\tilde{g}_{12}^2} \geq 1$, so

$$(\text{tr}\, g^\alpha(\mathbf{k}))^2 \geq 4 \det g^\alpha(\mathbf{k}), \tag{16}$$

with equality if and only if g^α is proportional to the identity matrix. Because the Berry curvature is algebraically independent of the components of the quantum metric, we can conclude that saturation of trace inequality (12) implies saturation of the determinant inequality (13). In physical terms, a constant curvature and metric that saturate the determinant inequality imply the closure of a modified Girvin-MacDonald-Platzman (GMP) algebra; saturation of the trace inequality then corresponds to the stronger condition that the algebra of band projected density operators is not only isomorphic to the GMP algebra, but identical to it.

Supplementary Note 3: Two-band models

By adding suitably chosen exponentially-localized couplings, any two-band Hamiltonian may be brought to a band-flattened form parameterized by a unit 3-vector $H_{\text{flat}}(\mathbf{k}) = -\widehat{\mathbf{n}}(\mathbf{k}) \cdot \sigma$, where $\sigma = (\sigma_1, \sigma_2, \sigma_3)$ are the three Pauli matrices. The eigenvectors of the original and flattened Hamiltonians are identical (by construction) and can be obtained analytically:

$$u^{\pm}(\mathbf{k}) = \frac{1}{\sqrt{2(1 \mp \widehat{\mathbf{n}}_3)}} \begin{pmatrix} \widehat{\mathbf{n}}_3 \mp 1 \\ \widehat{\mathbf{n}}_1 + i\widehat{\mathbf{n}}_2 \end{pmatrix}. \tag{17}$$

Using this expression gives the Berry curvature and quantum metric of the lower band as

$$B = \tfrac{1}{2}\widehat{\mathbf{n}} \cdot \partial_x \widehat{\mathbf{n}} \times \partial_y \widehat{\mathbf{n}}; \tag{18}$$

$$g_{\mu\nu} = \tfrac{1}{4}\partial_\mu \widehat{\mathbf{n}} \cdot \partial_\nu \widehat{\mathbf{n}}, \tag{19}$$

since $\widehat{\mathbf{n}} \cdot \partial_\mu \widehat{\mathbf{n}} = 0$. Using this and standard identities relating multiple dot and cross products, it follows that

$$g_{xx}(\mathbf{k}) g_{yy}(\mathbf{k}) - g_{xy}(\mathbf{k})^2 - \frac{1}{4} B(\mathbf{k})^2 = 0 : \tag{20}$$

the determinant condition $\det g^\alpha(\mathbf{k}) - B_\alpha(\mathbf{k})^2/4 = 0$ is necessarily satisfied for any two-band model. As our results with the kagomé and ruby lattice models show, this ceases to be the case for models having more than two bands.

Supplementary Note 4: Scaling of gaps with the number of bands

Fractional Chern insulators (FCIs) exhibit states which may be thought of as discretizations of continuum fractional quantum Hall (FQH) states, in that they have identical topological and long-wavelength properties (see, e.g., Supplementary Ref. 10). To that end, consider a continuum position-space wavefunction $\psi(\mathbf{r})$ which is discretized to set of values ψ_b in a tight-binding model with $\mathcal{N}$ sites per unit cell. The normalization conditions on the corresponding Bloch functions are

$$\int_{\text{UC}} d\mathbf{r}\, |u_{\mathbf{k}}(\mathbf{r})|^2 = \sum_{b=1}^{\mathcal{N}} |u_{\mathbf{k},b}|^2 = 1, \tag{21}$$

for any $\mathbf{k}$, where the integral is taken over a unit cell of the lattice. In the limit of large $\mathcal{N}$, we may assume that $u_{\mathbf{k}}(\mathbf{r})$ at $\mathbf{r} = \mathbf{d}_b$ is proportional to the discretized value $u_{\mathbf{k},b}$. Approximating the continuum normalization integral by a sum introduces a factor of $1/\mathcal{N}$ from the integration measure, which is compensated by the scaling

$$u_{\mathbf{k}}(\mathbf{d}_b) \sim \sqrt{\mathcal{N}} u_{\mathbf{k},b}. \tag{22}$$

Now consider the matrix elements of the delta-function interaction employed in the main text to stabilize the bosonic Laughlin state. In the continuum, these are

$$\begin{aligned}\langle \mathbf{k}_3\mathbf{k}_4|\widehat{V}|\mathbf{k}_1\mathbf{k}_2\rangle &= V \int d\mathbf{r}\, u^*_{\mathbf{k}_3}(\mathbf{r})u^*_{\mathbf{k}_4}(\mathbf{r})u_{\mathbf{k}_1}(\mathbf{r})u_{\mathbf{k}_2}(\mathbf{r}) \\ &\sim \frac{V}{\mathcal{N}} \sum_b u^*_{\mathbf{k}_3}(\mathbf{d}_b)u^*_{\mathbf{k}_4}(\mathbf{d}_b)u_{\mathbf{k}_1}(\mathbf{d}_b)u_{\mathbf{k}_2}(\mathbf{d}_b),\end{aligned}$$

times a momentum-conserving δ-function. Comparing this with an on-site interaction in the discretized model

$$V_{\text{disc}} \sum_b u^*_{\mathbf{k}_3,b}u^*_{\mathbf{k}_4,b}u_{\mathbf{k}_1,b}u_{\mathbf{k}_2,b}$$

and using (22) shows that the discretized interaction strength should be scaled as

$$V_{\text{disc}} \sim V\mathcal{N} \qquad (\delta\text{-function}). \tag{23}$$

This means that, given gaps which have been obtained for the FCI Laughlin state in two lattice models A, B, the quantities which should be compared are $\mathcal{N}_A\Delta_A$ and $\mathcal{N}_B\Delta_B$ (assuming that the single-particle dispersion has been flattened; i.e. that the gap is only set by the interaction term.) Similar considerations show that for the three-body delta-function interaction used to stabilize the Moore-Read state, the leading

scaling should be $V_{\text{disc}} \sim V\mathcal{N}^2$.

We note that the above argument is not as simple as it may appear: we're implicitly assuming $\psi(\mathbf{r})$ has the character of a low-lying eigenstate — more specifically, that it has support over almost all tight-binding orbitals. This is the case when $u_{\mathbf{k},b}$ is a randomly chosen vector from the $\mathcal{N}$-dimensional band Hilbert space, but one can easily construct counterexamples violating this assumption: for example, let lattice model B (with $\mathcal{N}_B > \mathcal{N}_A$) have a block-diagonal kinetic term, the first block of which is identical to the kinetic term of model A and the second block of which describes additional, trivial "spectator" orbitals with very high occupation energies. The low-lying bands $u^B_{\mathbf{k},b}$ will have vanishing amplitude on these spectator orbitals, and will numerically be identical to the corresponding eigenfunctions $u^A_{\mathbf{k},b}$ of model A, with zeros appended. In this scenario, the eigenfunction scaling assumption (22) is violated, and the interaction strength (and gap size) would *not* scale with the number of spectator orbitals.

The scaling argument can be placed on a more rigorous footing in the context of the Hofstadter model[11] in the limit of small flux per plaquette $\phi = 1/N$, which offers a sequence of lattice models (with N bands) which converges to the continuum FQHE in the $N \to \infty$ limit. In this case, the scalings (22), (23) are numerically observed to be obeyed to high accuracy.[12] For the case of the FCI models studied in this work, we know the ground states of different models lie in the same universality class as the continuum FQHE state (as can be determined by topological order, done here by entanglement spectrum counting), but we do not expect, e.g., the ruby lattice model ground state to be an interpolation of the kagomé lattice model ground state. The fact that we observe the scaled gaps to be so close to each other provides an *a posteriori* confirmation of this argument's validity even for the relatively small values of $\mathcal{N} = 2, 3, 6$.

Supplementary Note 5: Expansion of band-geometric quantities in parameter space

The Berry curvature and quantum metric are defined in terms of the derivatives of a wavefunction over some parameter manifold. For both numerical and theoretical purposes, it becomes convenient to recast these expressions in terms of derivatives of the Hamiltonian itself, rather than its eigenfunctions, since ordinarily only the former is known analytically. This, of course, is not a new observation.[13]

In order to avoid overall phase ambiguities and the necessity of using multiple charts to cover the Brillouin zone (BZ) in topologically non-trivial situations, it's preferable to work with the occupied band projector $P_\alpha = |\mathbf{k}, \alpha\rangle\langle\mathbf{k}, \alpha|$, instead of the eigenfunction $|\mathbf{k}, \alpha\rangle$ itself. In these terms, the Feynman-

Hellman theorems are

$$\partial_\lambda E_\alpha = \text{tr}\, P_\alpha\, \partial_\lambda H; \tag{24}$$

$$\partial_\lambda P_\alpha = R_\alpha\, \partial_\lambda H\, P_\alpha + P_\alpha\, \partial_\lambda H\, R_\alpha, \tag{25}$$

where the projected resolvent operator R_α is

$$R_\alpha = \sum_{\beta\neq\alpha} \frac{|\beta\rangle\langle\beta|}{E_\alpha - E_\beta} = \frac{1 - P_\alpha}{E_\alpha - H}. \tag{26}$$

Equations (24) and (25) hold for any parameter λ upon which the Hamiltonian smoothly depends, and they are valid for arbitrary values of λ and hence may be further differentiated.

Assuming no additional degeneracies, $(E_\alpha - H)$ may be inverted in the subspace orthogonal to $|\mathbf{k}, \alpha\rangle$, and

$$\partial_\lambda R_\alpha = -R_\alpha^2\, \partial_\lambda H\, P_\alpha - P_\alpha\, \partial_\lambda H\, R_\alpha^2 - R_\alpha \partial_\lambda (E_\alpha - H)\, R_\alpha. \tag{27}$$

The relations (24), (25) and (27) then form a closed system and may be iterated to any order to develop the Taylor expansion of band-geometric quantities.

For example, the Berry curvature and quantum metric for a single occupied Chern band are[14,15]

$$B_\alpha = 2\,\text{Im tr}\, P_\alpha\, \partial_y H\, R_\alpha^2\, \partial_x H, \tag{28}$$

$$g_{\mu\nu}^\alpha = \text{Re tr}\, P_\alpha\, \partial_\mu H\, R_\alpha^2\, \partial_\nu H. \tag{29}$$

The derivative of the Berry curvature with respect to a coupling λ follows from a straightforward computation as

$$\begin{aligned} \partial_\lambda B_\alpha = 2\,\text{Im tr}\, P_\alpha \big\{ &-2(\partial_\lambda E_\alpha)\partial_y H\, R_\alpha^3\, \partial_x H + \partial_y H\, R_\alpha^2\, \partial_{x,\lambda} H + \partial_{y,\lambda} H\, R_\alpha^2\, \partial_x H \\ &+ \partial_y H\, R_\alpha (\partial_\lambda H\, R_\alpha + R_\alpha\, \partial_\lambda H) R_\alpha\, \partial_x H \\ &+ \big[\big(\partial_\lambda H\, R_\alpha\, \partial_y H\, R_\alpha^2\, \partial_x H + (\partial_x E_\alpha) \partial_\lambda H\, R_\alpha^3\, \partial_y H \big) - (x \leftrightarrow y) \big] \big\}. \end{aligned} \tag{30}$$

Other quantities such as the Hessian $\partial_\lambda \partial_{\lambda'} B_\alpha$ may be calculated in a similar manner, although the algebra rapidly becomes tedious. These may, in turn, be used to find the variation in Brillouin zone-averaged quantities with respect to couplings in the Hamiltonian; for example, denoting by $\langle \cdots \rangle$ the Brillouin zone

average,

$$c_1 = \frac{A_{BZ}}{2\pi}\langle B\rangle, \tag{31}$$

$$\sigma_B = \frac{A_{BZ}}{2\pi}\sqrt{\langle B^2\rangle - \langle B\rangle^2}, \tag{32}$$

$$\text{and so } \partial_\lambda \sigma_B = \frac{(A_{BZ}/2\pi)^2}{\sigma_B}\langle B\partial_\lambda B\rangle. \tag{33}$$

Here we've made use of the fact that $\partial_\lambda\langle B\rangle = 0$ while we remain in the same topological phase, which can easily be checked during numerical computations. Using (30) in (33) then permits us to rapidly perform a steepest-descent minimization of Berry curvature fluctuations, even in a high-dimensional parameter space.

SUPPLEMENTARY REFERENCES

[1] Wu, Y.-L., Bernevig, B. A. & Regnault, N. Zoology of fractional Chern insulators. *Phys. Rev. B* **85**, 075116 (2012).

[2] Zak, J. Berry's phase for energy bands in solids. *Phys. Rev. Lett.* **62**, 2747–2750 (1989).

[3] Zak, J. Berry's geometrical phase for noncyclic Hamiltonians. *Europhys. Lett.* **9**, 615–620 (1989).

[4] Sundaram, G. & Niu, Q. Wave-packet dynamics in slowly perturbed crystals: Gradient corrections and Berry-phase effects. *Phys. Rev. B* **59**, 14915–14925 (1999).

[5] Karplus, R. & Luttinger, J. Hall effect in ferromagnetics. *Phys. Rev.* **95**, 1154–1160 (1954).

[6] Xiao, D., Chang, M.-C. & Niu, Q. Berry phase effects on electronic properties. *Rev. Mod. Phys.* **82**, 1959–2007 (2010).

[7] Duca, L. *et al.* An Aharonov-Bohm interferometer for determining Bloch band topology. *Science* **347**, 288–292 (2015).

[8] Lee, C. H., Thomale, R. & Qi, X.-L. Pseudopotential formalism for fractional Chern insulators. *Phys. Rev. B* **88**, 035101 (2013).

[9] Roy, R. Band geometry of fractional topological insulators. *Phys. Rev. B* **90**, 165139 (2014).

[10] Regnault, N. & Bernevig, B. A. Fractional Chern insulator. *Phys. Rev. X* **1**, 021014 (2011).

[11] Hofstadter, D. Energy levels and wave functions of Bloch electrons in rational and irrational magnetic fields. *Phys. Rev. B* **14**, 2239–2249 (1976).

[12] Bauer, D., Jackson, T. & Roy, R. Quantum geometry and stability of the fractional quantum Hall effect in the Hofstadter model. Preprint at http://arxiv.org/abs/1504.07185 (2015).

[13] Berry, M. V. Quantal phase factors accompanying adiabatic changes. *Proc. R. Soc. A* **392**, 45–57 (1984).

[14] Avron, J. & Seiler, R. Quantization of the Hall conductance for general, multiparticle Schrödinger Hamiltonians. *Phys. Rev. Lett.* **54**, 259–262 (1985).

[15] Campos Venuti, L. & Zanardi, P. Quantum critical scaling of the geometric tensors. *Phys. Rev. Lett.* **99**, 095701 (2007).
